# Sex-Dependent RNA Editing and *N6*-adenosine RNA Methylation Profiling in the Gonads of a Fish, the Olive Flounder (*Paralichthys olivaceus*)

**DOI:** 10.3389/fcell.2020.00751

**Published:** 2020-08-05

**Authors:** Lijuan Wang, Zhihao Wu, Congcong Zou, Shaoshuai Liang, Yuxia Zou, Yan Liu, Feng You

**Affiliations:** ^1^Key Laboratory of Experimental Marine Biology, Center for Ocean Mega-Science, Institute of Oceanology, Chinese Academy of Sciences, Qingdao, China; ^2^Laboratory for Marine Biology and Biotechnology, Pilot National Laboratory for Marine Science and Technology (Qingdao), Qingdao, China; ^3^College of Earth and Planetary Sciences, University of Chinese Academy of Sciences, Beijing, China

**Keywords:** RNA editing, *N6*-methyladenosine, ovary, testis, *Paralichthys olivaceus*

## Abstract

Adenosine-to-inosine (A-to-I) editing and *N6*-methyladenosine (m6A) are two of the most abundant RNA modifications. Here, we examined the characteristics of the RNA editing and transcriptome-wide m6A modification profile in the gonads of the olive flounder, *Paralichthys olivaceus*, an important maricultured fish in Asia. The gonadal differentiation and development of the flounder are controlled by genetic as well as environmental factors, and the epigenetic mechanism may play an important role. In total, 742 RNA editing events were identified, 459 of which caused A to I conversion. Most A-to-I sites were located in 3′UTRs, while 61 were detected in coding regions (CDs). The number of editing sites in the testis was higher than that in the ovary. Transcriptome-wide analyses showed that more than one-half of the transcribed genes presented an m6A modification in the flounder gonads, and approximately 60% of the differentially expressed genes (DEGs) between the testis and ovary appeared to be negatively correlated with m6A methylation enrichment. Further analyses revealed that the mRNA expression of some sex-related genes (e.g., *dmrt1* and *amh*) in the gonads may be regulated by changes in mRNA m6A enrichment. Functional enrichment analysis indicated that the RNA editing and m6A modifications were enriched in several canonical pathways (e.g., Wnt and MAPK signaling pathways) in fish gonads and in some pathways whose roles have not been investigated in relation to fish sex differentiation and gonadal development (e.g., PPAR and RNA degradation pathways). There were 125 genes that were modified by both A-to-I editing and m6A, but the two types of modifications mostly occurred at different sites. Our results suggested that the presence of sex-specific RNA modifications may be involved in the regulation of gonadal development and gametogenesis.

## Introduction

RNA transfers genetic information from DNA to protein and occupies a central position in all cellular processes. RNA modifications provide a rapid direct way to regulate gene expression. To date, more than 150 chemical modifications have been identified in eukaryotic mRNA (Helm and Motorin, 2017). Among these RNA modifications, adenosine-to-inosine (A-to-I) editing and N6-methyladenosines (m6A) are two of the most abundant. Both of these RNA modifications occur on A bases, but there is a negative correlation between them (Xiang et al., 2018). RNA editing is an enzyme-mediated post/cotranscriptional mechanism that alters the nucleotide composition of an RNA sequence without affecting the encoding DNA sequence (Farajollahi and Maas, 2010; Nishikura, 2016). This modification leads to differences between the transcript sequence and its template DNA, thereby recoding the open reading frame, affecting alternative splicing, and influencing RNA stability (Heale et al., 2010; Nishikura, 2016; Tajaddod et al., 2016). A-to-I editing is the predominant type of RNA editing and is catalyzed by Adenosine Deaminases Acting on RNA (ADARs) (Nishikura, 2016). ADAR enzymes (ADAR1, ADAR2, and ADAR3) bind to double-stranded RNAs (dsRNAs) and deaminate A to I. As the inosine (I) is generally read as guanosine (G) with the cellular machinery in the translation event, A-to-I substitution leads to A-to-G transition in the edited substrate (Nishikura, 2010). Thus, A-to-I editing is also named A-to-G editing (Bazak et al., 2014; Zhang et al., 2019). ADARs are essential for normal development, and knockout or homozygous null mutations in ADAR genes cause lethality in mice (*Mus musculus*) (Higuchi et al., 2000; Wang et al., 2000). RNA editing studies are being widely implemented in mammals by comparing matched RNA and DNA sequencing data (Tan et al., 2017), and most of these studies focus on the associations with diseases ranging from cancer to neurological diseases (Galeano et al., 2012; Slotkin and Nishikura, 2013; Wang et al., 2017). Some RNA editing events are tissue specific (Tao et al., 2012), but the available knowledge about RNA editing in gonads is very limited. In mouse testis, *adar1* and *adar2* genes are expressed in both Sertoli cells and the germline in a cell-type-dependent manner during germ cell development (Snyder et al., 2017), and 2012 genes exhibiting editing events in germ cells during spermatogenesis have been detected (Wang et al., 2019). However, the functions of RNA editing in gonads are still unknown.

*N6*-methyladenosine (m6A) was the first RNA modification shown to be reversible in eukaryotic messenger RNA. This modification is catalyzed by methyltransferases known as “writers” composed of a core complex consisting of Methyltransferase-like 3 (*mettl3*), *mettl14*, Wilms tumor 1-associating protein (*wtap*), and other subunits; can be reversed by “erasers” including fat mass and obesity-associated protein (*fto*) and AlkB homolog 5 (*alkbh5*); and can be recognized by “readers” such as YTH domain-containing proteins (*ythdc1-2* and *ythdf1-3*) (Roundtree et al., 2017). It is an essential regulator of gene expression and can regulate the metabolism and processing of transcripts (Frye et al., 2018). The functions of m6A depend on its recognition by specific reader proteins that bind to methylated mRNAs. For example, in the nucleus, *ythdc1* regulates splicing by binding to pre-RNAs, while in the cytoplasm, *ythdf2* targets the transcripts for degradation, and *ythdf1, ythdf3*, and *ythdc2* promote the translation of methylated mRNA (Patil et al., 2017; Liao et al., 2018). M6A plays critical roles in a variety of biological processes such as the maintenance of mRNA stability, miRNA maturation (Alarcón et al., 2015), stemness (Batista et al., 2014), circadian rhythms (Jean-Michel et al., 2013), and disease (Du et al., 2019). Increasing evidence has revealed crucial roles for m6A modification during sex determination, gonadal development, and gametogenesis in various taxa. In *Drosophila*, *Ime4*, the *mettl3* homolog, can regulate sex by facilitating the alternative splicing of sex lethal (*sxl*), an important regulator of dosage compensation and the key sex determination factor (Haussmann et al., 2016; Lence et al., 2016). Other works have shown that m6A is involved in mammalian fertility and spermatogenesis (Hsu et al., 2017; Xu et al., 2017), amphibious oocyte meiotic maturation (Qi et al., 2016), and avian follicle selection (Fan et al., 2019). Deletion of *mettl3*, *mettl14*, *fto*, *alkbh5*, *ythdf2*, or *ythdc2* leads to impaired fertility and defects in gametogenesis (Frye et al., 2018; Huang and Yin, 2018).

Fish are the most abundant vertebrates on earth, and their sex determination mechanisms exhibit extraordinary plasticity, diversity, and adaptability (Mei and Gui, 2015). To date, master sex-determination genes have been identified for only a few fish species, and fish sex determination and differentiation can be affected by the genetic system, exogenous hormones, and environmental factors (Capel, 2017). Epigenetic mechanisms provide fish with the ability to integrate genomic and environmental information for the development of a particular sex phenotype. Several steroidogenic enzymes and transcription factors are likely to be epigenetically regulated (Piferrer, 2018), including the important steroidogenic enzyme cytochrome P450 aromatase (encoded by *cyp19a*), but the understanding of the mechanism is limited to the DNA methylation of these genes’ promoters. As mentioned above, RNA modifications provide additional mechanisms of gene expression regulation without a DNA sequence change (Li et al., 2017) and could provide new insight into fish sex determination. Recently, m6A and RNA editing have been found to play important roles in the development of the gonads and other tissues. However, scant information is available for fish gonads barring one report addressing m6A in zebrafish (*Danio rerio*) showing that knockout of the m6A methyltransferase “writer” *mettl3* led to a defect in sperm maturation and reduced sperm motility as well as a significant decrease in 11-ketotestosterone (11-KT) and 17β-estradiol (E2) levels (Xia et al., 2018). There has been no information about RNA editing in fish gonads reported to date. Therefore, these two RNA modifications remain largely unknown in fish gonads. The distribution patterns and functional relevance of these two modifications should be studied as an initial step to reveal the roles of m6A and RNA editing in gonads.

The olive flounder, *Paralichthys olivaceus*, is a commercially important marine fish in China, Japan, and Korea. It exhibits an XX (female)/XY (male) sex determination mechanism (Yamamoto, 1999), but its sex determination and gonadal development are also influenced by environmental factors, especially temperature (Sun et al., 2010). Previous studies have proven that the promoter DNA methylation levels of some steroidogenic-related genes are different between the ovary and testis (Wen et al., 2014; Si et al., 2016; Fan et al., 2017), which indicates that sex determination and gonadal development could be regulated by epigenetic mechanisms in this species. Similar to most fish, there is no available information on RNA modifications of the flounder gonad. Gynogenesis in fish can rapidly produce offspring with increased genetic similarity and is an efficient way of breeding (Yamamoto, 1999; You et al., 2001). The genotype of gynogenetic flounders is XX (female), but the phenotypic sex can be reversed to male through changes in environmental factors. Thus, this species could provide an appropriate model for studying sex determination and maintenance of phenotypic sex in fish. In this study, we systematically characterized the RNA editome based on high-throughput RNA sequencing data and whole-genome sequencing data for the gonads and muscle of the wildtype and meiogynogenetic diploid flounders. Additionally, RNA methylation profiling was performed via methylated RNA immunoprecipitation sequencing (MeRIP-Seq) to examine the differences in m6A modifications between the ovary and testis. This RNA modification profiling of the male and female flounder gonads adequately elucidates the sexual molecular mechanism and may provide new insights into the molecular mechanisms underlying the gene regulatory network controlling sex determination and subsequent maintenance of phenotypic sex in fish.

## Results

### Developmental Stage of the Flounder Gonadal Samples

**FIGURE 1:**
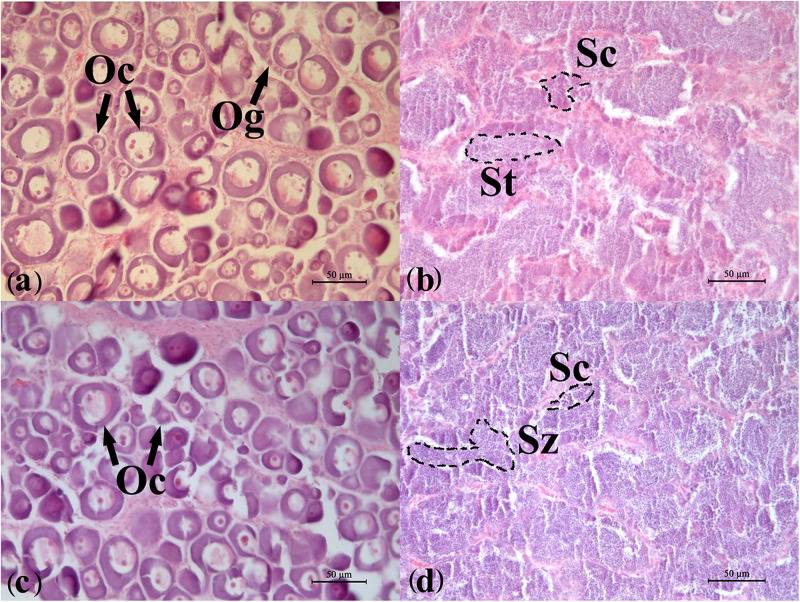
Developmental stages of the flounder gonads determined by histology. **(a)** Ovary of wildtype female (WF); **(b)** testis of wildtype male (WM); **(c)** ovary of gynogenetic female (GF); **(d)** testis of gynogenetic male (GM). Oc, oocyte; Og, oogonium; St, spermatid; Sc, spermatocyte; Sz, spermatozoon. Scale bar, 50 μm.

According to the HE results from the gonadal histological sections (Figure 1), the developmental stage of all the ovaries used in this study was stage II, which included a large number of oocytes of phase II. In oocytes, one or numerous large nucleoli were at the periphery of the nucleus, and the nucleus was evident. Follicular cells appeared outside of the cell membrane of a small number of oocytes. The sizes of oocytes were bigger than those of oogonia, and cytoplasm were bluish violet because of basophilia. The developmental stage of the testes used was IV-V. The primary spermatocytes, secondary spermatocytes, and spermatids were all observed, which showed typical characteristics of stage IV of the testis. In the meanwhile, more mature spermatozoa were also found and spermatogonia were almost invisible in the sections showing the testis were developing into stage V.

### Genome-Wide Analysis of RNA Editing Sites in the Flounder Gonads

#### Identification of RNA Editing Sites

Candidate RNA editing sites at the transcriptome-wide level were detected by RNA sequencing in the gonads and matched DNA sequencing in the muscle from the WM, WF, GM, and GF samples. A summary of the sequencing results is provided in Table 1. Approximately 96.76% of the 520.34 million pass-filter reads obtained from DNA sequencing were successfully aligned to the flounder reference genome. After quality trimming, 49.79 million RNA reads were generated from each sample on average, with an average mapping rate of 81.98%. Possible RNA editing events were detected by comparing matched RNA-seq and DNA-seq data. A site was considered to be a potential editing site when it was detected in at least two individuals. Combining these approaches, a total of 742 editing sites were identified from the RNA-seq data of the gonads (Supplementary Table S1).

**TABLE 1:** Summary of the flounder gonadal DNA and RNA sequencing and mapping results.

Sample name	Tissue type	DNA-seq			RNA-seq		
		Number of clean read	Number of mapped read	Mapping rate of read (%)	Number of clean read	Number of mapped read	Mapping rate of read (%)
WF1	Wildtype female	42,499,992	41,036,301	96.56	50,388,270	45,037,312	89.38
WF2		41,854,356	40,516,586	96.80	50,413,680	45,148,923	89.56
WF3		40,967,184	39,639,589	96.76	47,206,102	42,234,008	89.47
WM1	Wildtype male	45,236,680	43,834,775	96.90	53,008,080	39,432,748	74.39
WM2		43,638,032	42,257,109	96.84	50,379,920	36,021,023	71.50
WM3		46,227,002	44,764,853	96.84	51,907,244	36,984,811	71.25
GF1	Gynogenetic female	48,716,326	47,148,981	96.78	45,650,496	40,712,533	89.18
GF2		36,463,686	35,197,766	96.53	54,144,024	48,435,930	89.46
GF3		43,670,642	42,253,975	96.76	54,144,462	48,295,002	89.20
GM1	Gynogenetic male	45,329,746	43,806,795	96.64	46,719,724	36,519,413	78.17
GM2		39,900,490	38,640,720	96.84	46,268,008	36,508,023	78.91
GM3		45,840,636	44,376,521	96.81	47,247,914	34,600,456	73.23

#### Characterization of the Flounder Gonadal RNA Editing Sites

**FIGURE 2:**
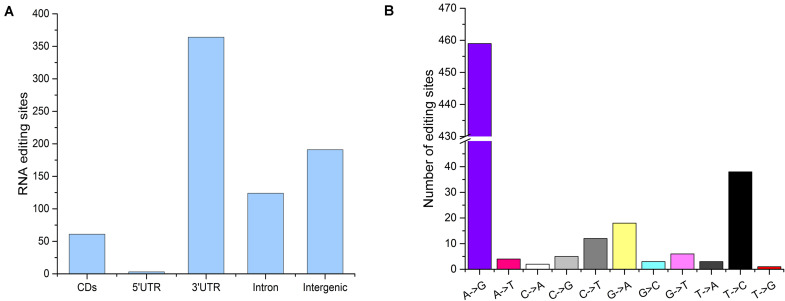
Characteristics of the editome of the flounder gonads. **(A)** Distribution of the genomic locations of the identified editing sites. **(B)** Distribution of RNA editing types.

The distribution of the genomic locations of A-to-I, C-to-U, and non-canonical editing sites is shown in Figure 2A. Among all editing sites, a large fraction (364, 49.06%) were located in the non-coding 3′UTRs, and 61 (8.22%) sites were resided in coding sequences, 21 of which resulted in an amino acid change (Figure 2A). All intergenic-associated sites were near annotated genes, and some might represent extended 5′UTRs or 3′UTRs. Among the 742 detected editing sites, 551 editing events were located within annotated genes and were used for further analysis. All 12 possible base substitutions resulting from RNA editing except for A-to-C substitution were detected in this study (Figure 2B). A-to-G substitution was found to be the most common type, and there were a total of 459 A-to-G editing sites detected in the flounder gonads, accounting for up to 83.30% of the identified RNA editing sites. C-to-U substitutions accounted for approximately 3% of the sites (12 sites). Furthermore, most of the edited genes were edited in only one type of genic regions, such as an intron, CDS or 3′UTR.

#### Different RNA Editing Distributions in the Flounder Ovary and Testis

**FIGURE 3:**
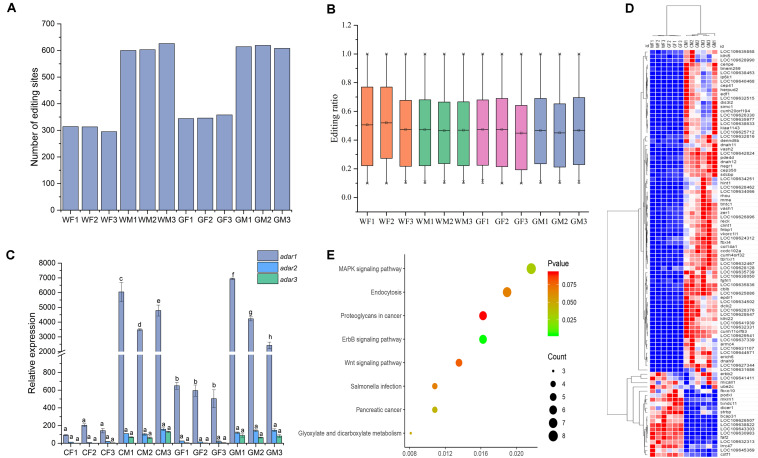
Signatures of editing sites in the flounder ovary and testis. **(A)** Total number of editing sites in the 12 flounder gonads. **(B)** Distribution of the RNA editing ratios across the 12 gonadal tissues. **(C)** ADAR enzyme expression across gonadal tissues determined by using qPCR. All data represent the mean values of three biological replicates. Different lowercase letters indicate significance differences between samples according to *post hoc* Duncan’s multiple range test (*p* < 0.05). **(D)** Relative expression of genes with different editing levels in the 12 gonadal tissues. The expression of genes is color coded from low (blue) to high (red). **(E)** Pathway classifications of differentially expressed genes according to KEGG.

The RNA editing sites varied among the samples. Overall, number of the RNA editing sites in the testis was higher than that in the ovary. Average numbers of the editing events in the gonads of the WM, WF, GM, and GF samples were 610, 307, 614, and 349, respectively. The sample with the highest number of the edited sites was WM3 (*n* = 626, Figure 3A), while the sample with the lowest number was WF3 (*n* = 295, Figure 3A). However, the average RNA editing ratios were similar across different groups; in the WF, WM, GF, and GM samples, the ratios were 0.50, 0.47, 0.46, and 0.46, respectively (Figure 3B). Most of the mean editing ratios between the samples were not significantly different (Supplementary Table S2).

A-to-I editing catalyzed with ADAR enzymes was the most abundant editing type in the flounder gonads. The expression levels of the ADAR enzyme genes (*adar1, adar2*, and *adar3*) in the gonads were also analyzed to investigate whether the differences in the RNA editing pattern were related to the expression of the *adar* genes. The results determined by RNA-seq and qPCR showed that the *adars* presented higher expression in the testis than in the ovary, which was in accordance with the higher number of the RNA editing sites in the testis (Figure 3C). As shown in Figure 3C, there was a relationship between the *adar* expression and RNA editing patterns in different samples. Overall, we found a trend of a positive correlation (Pearson correlation = 0.948, *p* < 0.01) between the expression of the *adar1* and tissue-specific RNA editing.

To explain the differential or sex-specific RNA editing sites in the flounder gonads, the expression of the genes edited in the ovary and testis was investigated. Among the 255 edited genes, 97 and 93 genes were differentially expressed in the WF vs. WM and GF vs. GM comparisons, respectively. Most of the genes expressed in the testis showed higher expression than those expressed in the ovary (Figure 3D). Within the detected editing sites, the editing ratios of 299 sites were different between the ovary and testis, and most editing events occurred only in the testis. Some of these sex-differential editing genes were related to gonadal development and gametogenesis, such as Cullin-3 (*cul3*), SRSF protein kinase 2 (*srpk2*), and DAZ-associated protein 2 (*dazap2*). Among the 255 genes, 73 were mapped to KEGG pathways. These genes were mainly associated with the “ErbB signaling pathway,” “MAPK signaling pathway,” and “Salmonella infection” (Figure 3E). KEGG enrichment revealed that DEGs with different RNA editing levels were related to metabolic pathways, cell adhesion molecules (CAMs), and microRNAs in cancer.

### Transcriptome-Wide *N6*-methyladenosine Methylome Profiling of the Flounder Gonads

#### Transcriptome-Wide Detection of m6A Modifications

The MeRIP-Seq technique was applied to identify the target regions modified by m6A methylation in the flounder gonads. After filtering out low-quality data, 56,340,494, 59,429,986, 57,077,566, and 58,165,726 clean reads were obtained from the immunoprecipitated (IP) samples of the WM, WF, GM, and GF gonads, respectively. More than 50% of the IP reads uniquely mapped to the reference genome. Comparison of the m6A-IP data with the input data revealed 19,404 potential m6A peaks among 11,017 expressed genes in WF, 23,087 m6A peaks among 12,502 expressed genes in WM, 19,349 m6A peaks among 11,140 expressed genes in GF, and 22,084 m6A peaks among 12,176 expressed genes in GM. The information indicated that the flounder transcriptomes presented an estimated 1.793, 1.746, 1.702, and 1.637 m6A peaks per actively expressed transcript in WF, WM, GF, and GM, respectively.

**FIGURE 4:**
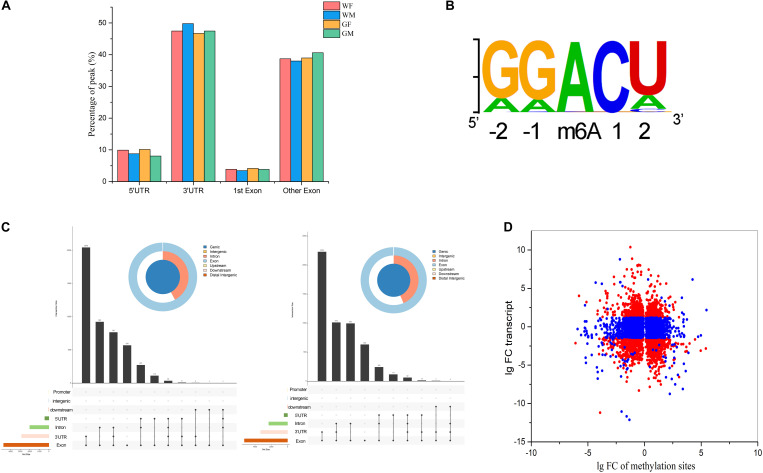
Signatures of m6A RNA methylation in the flounder gonads. **(A)** Distribution of the genomic locations of the identified m6A RNA methylation peaks. **(B)** Sequence preferences of m6A RNA methylation peaks. **(C)** Landscape of RNA editing positions with different peaks in the ovary and testis (left, WF vs. WM; right, GF vs. GM). **(D)** Fold changes in the relative expression and methylation sites of genes in the ovary and testis.

To understand the topological pattern of m6A methylation in the flounder gonadal transcriptome, the distribution profiles of m6A peaks in the entire transcriptomes of these gonads were also investigated. It was shown that m6A peaks were abundant in exons and 3′UTRs in both the ovary and testis, which showed higher abundance of these peaks than 5′UTRs (Figure 4A). Motifs that were enriched in regions surrounding m6A peaks were analyzed, and the results showed that the most frequent motif was GGACU (Figure 4B) in all groups, which was consistent with the classic consensus sequence of RRACH.

#### Different m6A Methylomes in the Flounder Ovary and Testis

The m6A marks in the transcripts of the ovary and testis were compared, and 4,741 and 5,313 m6A sites in 3,947 and 4,468 genes were found to be differentially methylated between the gonads of WF vs. WM and GF vs. GM, respectively. The different peaks were mostly present in CDs and 3′UTRs of the mRNAs but were also found in 5′UTRs (Figure 4C). Furthermore, the transcript expression levels of genes with differentially methylated m6A sites were analyzed. The association analysis between them was performed based on the RNA-Seq and MeIP-seq sequencing data. The results showed that among the 3,947 and 4,468 differentially methylated genes, the expression levels of 1,717 and 1,796 genes were significantly changed (Figure 4D). Among these DEGs that also exhibited differential m6A RNA methylation levels, 58% and 60% of the DEGs appeared to be negatively correlated with RNA methylation levels in the WF vs. WM and GF vs. GM comparisons, respectively.

**FIGURE 5:**
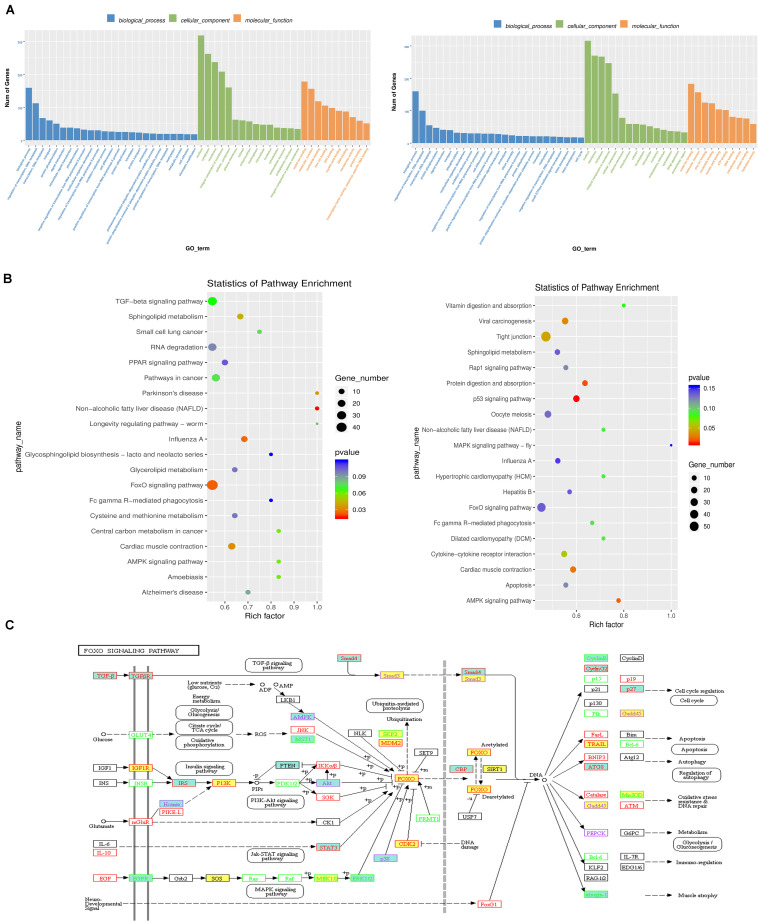
The regulatory role of the differential RNA methylation of genes showing differential expression between the flounder ovary and testis. **(A)** Gene ontology term (GO) assignment classes of DEGs (left, WF vs. WM; right, GF vs. GM). **(B)** KEGG pathway classifications of differentially expressed genes (left, WF vs. WM; right, GF vs. GM). **(C)** FoxO signaling pathway. Yellow background indicates m6A upregulated genes in the flounder ovary. Green background indicates m6A downregulated genes in the flounder ovary.

GO and KEGG enrichment analyses were conducted to decipher the regulatory roles of the differential RNA methylation of genes with different expression levels between the ovary and testis. The top results of the GO enrichment analysis are shown in a bar graph in Figure 5A and Supplementary Table S3, and the gene functional enrichment analysis showed that these genes were significantly enriched in cellular functional categories relevant to gonadal development and gametogenesis, such as “ciliary basal body,” “cilium,” “sequence-specific DNA binding,” and “Cul3-RING ubiquitin ligase complex.” The results of the KEGG pathway analysis of differentially methylated transcripts between the ovary and testis showed that the genes identified in the gonads of the WF vs. WM and GF vs. GM comparisons mapped to 272 and 260 pathways, respectively (Figure 5B and Supplementary Table S4). Among these pathways, several well-documented pathways associated with gonadal development and maintenance were identified as m6A targets controlled by m6A modifiers, including the “TGF-beta signaling pathway,” “Wnt signaling pathway,” “ovarian steroidogenesis,” “estrogen signaling pathway,” and “AMPK signaling pathway.” Notably, the FoxO signaling pathway was one of the most enriched pathways identified in both the WF vs. WM and GF vs. GM comparisons, implying a potential important role in gonadal development and gametogenesis. Within this pathway, 48 genes exhibited both differential m6A RNA methylation and gene expression levels (Figure 5C). In addition, some pathways that have not been previously investigated in depth during gonadal development in fish, such as the PPAR and RNA degradation pathways, were identified. PPAR showed high dissimilarity in both RNA methylation and gene expression between the testis and ovary. The significant changes in this pathway were driven by alterations in the expression levels of 12 related genes.

To identify differentially expressed transcripts with differential RNA methylation in the male and female gonads, the ovaries or testes of the wild and gynogenetic fish were grouped together as the female and male gonads to be compared. In total, 728 genes showed upregulation of both RNA methylation and expression, while 960 genes showed upregulation of RNA methylation and downregulation of expression, 673 genes showed downregulation of RNA methylation and upregulation of expression, and 618 genes showed downregulation of both methylation and expression. The RNA methylation levels and expression patterns of sex-related genes in fish or other vertebrates were checked. These genes have been reported as playing important roles in sex determination/differentiation, sex maintenance or gametogenesis. In total, 36 sex-related candidate genes were selected (Table 2), among which 18 were significantly more highly expressed in the testis compared to the ovary, while the others were more highly expressed in the ovary. Genes that are essential for testis differentiation and maintenance of male-specified germ cells in fishes and other vertebrates, such as doublesex and mab-3-related transcription factor 1 (*dmrt1*), sry box-containing gene 8 (*sox8*), sry box-containing gene 9 (*sox9*), anti-Müllerian hormone (*amh*), Wilms tumor 1 (*wt1*), deleted in azoospermia like (*dazl*), growth arrest and DNA damage-inducible gamma (*gadd45g*), and SIX homeobox 4 (*six4*), were identified and shown a male-biased expression pattern in the flounder. Sperm-associated antigen 5 (*spag5*), sperm-associated antigen 8 (*spag8*), sperm-associated antigen 17 (*spag17*), and spermatogenesis-associated protein 7 (*spata7*) were significantly more highly expressed in the testis compared to the ovary. Among the female-biased genes, some genes that have been proven to be involved in the regulation of ovarian differentiation, including bone morphogenetic protein 15 (*bmp15*), catenin beta 1 (*ctnnb1*), *foxo3*, and LIM homeobox 9 (*lhx9*), and genes encoding proteins implicated in oogenesis, such as zona pellucida sperm-binding protein 3 (*zp3*), were significantly highly expressed in the ovary.

**TABLE 2:** Sex-related genes with the m6A modification and fold change.

	Gene	m6A fold change	mRNA fold change	Chromosome	m6A position	Length
Male-biased gene	*amh*	1.60	0.00	chrNW_017859646.1	10,449,363–10,449,571	209
	*dazl*	1.21	0.20	chrNW_017859657.1	3,733,146–3,733,235	90
	*dmrt1*	1.66	0.10	chrNW_017860076.1	13,469–28,910	15,442
	*fgfrl1*	5.14	0.35	chrNW_017859641.1	2,265,936–2,266,593	658
	*hormad1*	1.75	0.70	chrNW_017859661.1	10,354,315–10,358,061	3,747
	*zte38*	2.66	0.03	chrNW_017859643.1	7,904,491–7,905,008	518
	*igf1r*	5.76	0.24	chrNW_017859645.1	11,739,635–11,740,234	600
	*igf1*	5.35	0.21	chrNW_017859644.1	5,267,377–5,267,886	510
	*wt1*	2.23	0.24	chrNW_017859672.1	1,079,804–1,080,162	359
	*six4*	3.78	0.18	chrNW_017859650.1	11,825,498–11,825,767	270
	*spag17*	2.57	0.06	chrNW_017859665.1	1,169,223–1,170,133	911
	*spag5*	2.02	0.21	chrNW_017859681.1	2,518,169–2,518,945	777
	*spag8*	1.95	0.32	chrNW_017859775.1	159,906–161,076	1,171
	*spata7*	2.72	0.14	chrNW_017859670.1	680,106–680,735	630
	*sycp3*	2.97	0.01	chrNW_017859642.1	3,155,125–3,157,121	1,997
	*sox8*	3.47	0.05	chrNW_017859684.1	531,402–532,330	929
	*sox9*	3.05	0.04	chrNW_017859684.1	278,963–279,499	537
	*gadd45g*	4.28	0.23	chrNW_017859693.1	324,524–325,031	508
Female-biased gene	*bmp15*	5.87	25.83	chrNW_017859764.1	334,982–336,653	1,672
	*err2*	7.20	5.88	chrNW_017860253.1	25,712–29,513	3,802
	*err*γ	5.62	2.08	chrNW_017859651.1	7,644,669–7,647,832	3,164
	*ctnnb1-like*	3.31	1.95	chrNW_017860320.1	18,668–22,561	3,894
	*foxo3*	10.00	2.82	chrNW_017859670.1	3,256,038–3,257,031	994
	*ctnnb1*	1.39	1.73	chrNW_017859658.1	5,463,788–5,463,848	61
	*cyp26b1*	13.9	3.05	chrNW_017859665.1	8,673,824–8,674,124	301
	*lhx9*	5.09	1.83	chrNW_017859671.1	3,017,250–3,021,737	4,488
	*ggnbp2*	1.45	3.63	chrNW_017859651.1	8,526,804–8,527,109	306
	*nfil3*	6.81	4.08	chrNW_017859647.1	1,039,435–1,039,854	420
	*nr0b1*	6.63	1.02	chrNW_017860693.1	5,061–5,475	415
	*spata13*	3.98	8.52	chrNW_017859653.1	9,162,467–9,162,946	480
	*spag7*	2.42	3.31	chrNW_017859651.1	3,686,278–3,688,083	1,806
	*sox4*	6.32	7.07	chrNW_017859658.1	10,493,432–10,494,656	1,225
	*sox11*	5.55	9.94	chrNW_017860424.1	3,354–3,977	624
	*zp3-like*	4.22	50.16	chrNW_017859650.1	8,480,795–8,481,453	659
	*zp3-like*	2.69	113.94	chrNW_017859643.1	13,044,125–13,044,650	526
	*zp3-like*	3.41	264.38	chrNW_017859650.1	6,855,597–6,856,408	812

**FIGURE 6:**
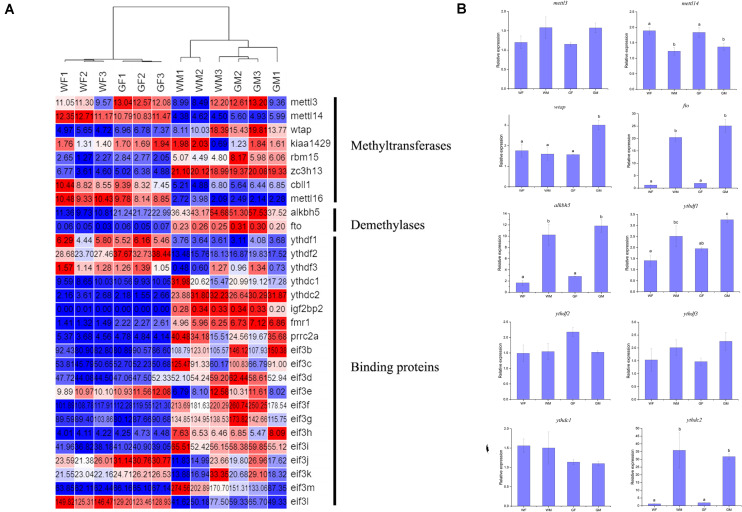
Relative expression of m6A methyltransferase genes in the flounder ovary and testis. **(A)** Heatmap analysis of key m6A methyltransferase genes in the flounder gonads based on the FPKM values from the RNA-seq data. Each row represents a gene listed on the right. Each column stands for a gonadal sample. The expression of genes is color coded from low (blue) to high (red). **(B)** qPCR validation of the RNA-seq data. All data represent the mean values of three biological replicates. Different lowercase letters indicate statistical significance between groups according to *post hoc* Duncan’s multiple range test (*p* < 0.05).

The genes encoding the methyltransferase ‘writer’ core complex (*mettl3, mettl14, wtap*, and other subunits, including Virilizer (*kiaa1429*), RNA-binding Motif Protein 15 (*rbm15*), and E3 ubiquitin ligase Casitas B-lineage lymphoma-transforming sequence-like protein 1 (*cbll1*, also known as HAKAI), were identified in the flounder transcriptome. In addition, the m6A “eraser” genes *fto* and *alkbh5*, the YTH domain-containing protein “reader” genes *ythdf1*, *ythdf2*, *ythdf3*, *ythdc1*, and *ythdc2*, and genes encoding other families of m6A readers, including eukaryotic initiation factor 3 (*eif3*), insulin-like growth factor 2 mRNA-binding protein 2 (*igf2bp2*), fragile X mental retardation 1 (*fmr1*), and Proline-rich coiled-coil 2A (*prrc2a*), were also detected in this study. The expression of *mettl3*, *mettl14*, *wtap*, *fto*, *alkbh5*, *ythdf1*, *ythdf2*, *ythdf3*, *ythdc1*, and *ythdc2* was investigated in the flounder ovary and testis with qPCR. The detected expression patterns of these genes were similar to those obtained by RNA-seq, indicating the reliability of the transcriptome expression analysis. The results also showed that the m6A “writer” *mettl14* was more highly abundant in the ovary than in the testis, while the “erasers” *fto* and *alkbh5* (*p* < 0.05) and the “readers” *ythdf1* and *ythdc2* exhibited especially high levels in the testis (*p* < 0.05, Figure 6).

### Comparison of Genes and Pathways Modified by A-to-I Editing and m6A Methylation

A total of 125 genes were modified by both m6A and A-to-I editing, and these genes were significantly enriched in signaling pathways regulating the pluripotency of stem cells, the Hippo signaling pathway, and the pancreatic cancer pathway. Thirty of these genes showed significantly different expression levels between the ovary and testis (Table 3). Both RNA modifications occurred at adenosines, but the RNA editing sites were mainly located in 3′UTRs, which did not coincide with the positions of the m6A modification. By comparing the pathways modified by A-to-I editing and m6A methylation, we found that the Wnt and MAPK signaling pathways were affected by both m6A and RNA editing modifications.

**TABLE 3:** Genes modified by both the m6A and A-to-I editing showing significantly different expression levels between the ovary and testis.

Gene	Fold change (WM/WF)	Log2 Fold change	*p*-value	Chromosome	m6A position	RNA editing position
*LOC109629541*	894.80	9.81	1.85E-64	chrNW_017859653.1	8,844,241–8,846,735	8,845,223
*vash2*	93.27	6.54	4.66E-15	chrNW_017860752.1	13,879–19,567	19,459
*ccdc102a*	9.48	3.24	1.28E-12	chrNW_017859645.1	17,757,967–17,758,146	17,758,510
*Spindlin1*	6.88	2.78	7.37E-11	chrNW_017860250.1	35,508–36,462	34,846
					36,671–37,419	35,048
						35,051
						35,056
*clint1*	6.67	2.74	9.22E-11	chrNW_017859641.1	13,967,203–13,969,370	13,961,651
						13,961,652
						13,961,654
						13,961,722
						13,962,084
						13,962,102
						13,962,104
						13,962,123
						13,962,127
						13,962,131
*fnbp1*	6.30	2.65	7.67E-10	chrNW_017859647.1	5,545,279–5,545,787	5,542,315
*vkorc1l1*	5.85	2.55	1.63E-09	chrNW_017859651.1	8,561,491–8,562,120	8,564,091
						8,564,933
						8,564,954
*LOC109635836*	5.91	2.56	3.27E-09	chrNW_017859668.1	4,980,665–4,981,052	4,972,623
						4,972,754
*mme*	6.95	2.80	5.42E-09	chrNW_017859649.1	7,532,318–7,533,076	7,532,061
						7,532,538
*LOC109634502*	24.99	4.64	1.35E-08	chrNW_017859664.1	8,722,040–8,722,129	8,722,140
*txndc11*	0.23	−2.13	2.78E-07	chrNW_017860014.1	16,905–18,562	19,882
					20,071–20,787	
					21,403–23,311	
*dclk2*	22.05	4.46	7.60E-07	chrNW_017859681.1	1,160,954–1,161,612	1,168,071
*edf1*	10.09	3.34	2.81E-06	chrNW_017859647.1	9,334,952–9,337,979	9,336,669
*LOC109635977*	8.52	3.09	1.19E-05	chrNW_017859642.1	12,403,967–12,404,656	12,404,291
						12,404,379
						12,404,380
						12,404,388
*LOC109632515*	8.51	3.09	6.77E-05	chrNW_017859659.1	3,028,743–3,029,043	3,029,305
*herpud2*	10.12	3.34	0.000257	chrNW_017859704.1	740,930–741,319	739,495
						739,497
*cep350*	2.61	1.39	0.00066	chrNW_017859646.1	13,799,152–13,799,498	13,811,820
						13,811,821
						13,811,828
						13,812,177
*ip6k1*	15.16	3.92	0.000845	chrNW_017859667.1	7,314,917–7,315,695	7,314,818
					7,315,845–7,316,402	
*fgfrl1*	2.60	1.38	0.001225	chrNW_017859641.1	2,266,115–2,266,623	2,262,929
*LOC109638633*	5.08	2.35	0.002465	chrNW_017859683.1	2,590,270–2,590,780	2,594,513
					2,591,066–2,591,282	
					2,592,236–2,592,386	
*cep41*	7.43	2.89	0.003062	chrNW_017859644.1	12,748,827–12,749,835	12,745,784
*LOC109634251*	2.20	1.14	0.004562	chrNW_017859663.1	9,556,053–9,556,262	9,559,090
					9,557,028–9,558,456	9,559,099
						9,559,100
*cstf1*	0.49	−1.03	0.007906	chrNW_017859652.1	13,217,381–13,220,892	13,216,762
						13,216,788
						13,216,789
						13,217,079
						13,217,100
						13,217,101
						13,217,116
*LOC109641411*	0.48	−1.07	0.008596	chrNW_017859850.1	187,907–188,593	187,707
*LOC109625712*	5.14	2.36	0.008792	chrNW_017859646.1	10,991,247–10,995,161	10,989,436
*tmem259*	2.90	1.54	0.010955	chrNW_017860128.1	32,083-32,382	31,869
					32,770–33,129	
*LOC109638453*	11.11	3.47	0.012789	chrNW_017859683.1	49,189–50,109	53,616
*fbxo10*	0.54	−0.89	0.030147	chrNW_017859678.1	1,069,311–1,069,759	1,069,888
						1,070,035
						1,070,046
*erbb2*	0.54	−0.90	0.031348	chrNW_017859827.1	6,928–7,108	4,302
						4,367
						4,451
*LOC109632816*	1.85	0.88	0.033611	chrNW_017859660.1	10,333,275–10,333,754	10,332,765
						10,332,766

Intriguingly, several ubiquitin-related genes, such as the critical gene of the Cullin-3-based E3 ubiquitin ligase complex, *cullin3*, were regulated by RNA editing, while some GO terms related to ubiquitin, “cilium,” and the “Cul3-RING ubiquitin ligase complex” were enriched in the DEGs with different m6A modifications. The E3 ubiquitin ligase HAKAI, one of the core constituents of the plant m6A writer complex, was also identified and shown female-biased expression.

### Comparison of Gene Expression Profiles in the Flounder Ovary and Testis

**FIGURE 7:**
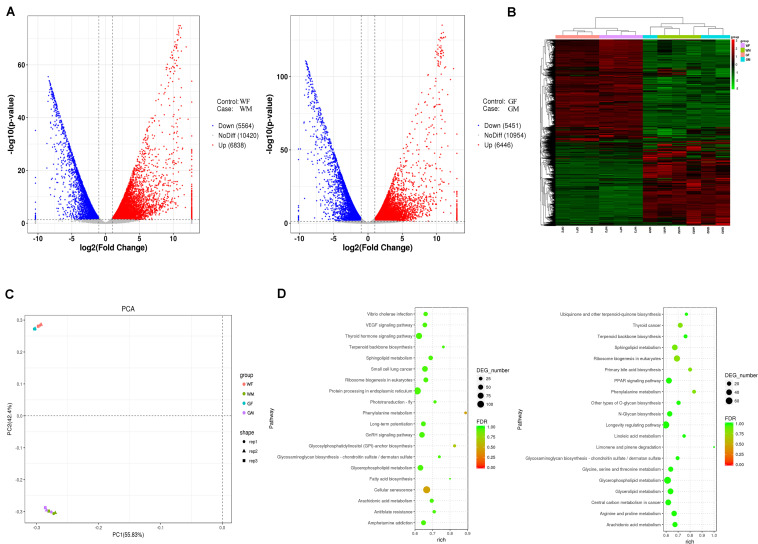
Gene expression profiles of the flounder ovary and testis. **(A)** Volcano plot of differentially expressed genes between the ovary and testis (left, WF vs. WM; right, GF vs. GM). **(B)** Heatmaps showing the global gene expression profiles of the flounder ovary and testis. Each row represents a gene. Each column represents a gonadal sample. The expression of genes is color coded from low (green) to high (red). **(C)** PCA plots of the gonadal samples. **(D)** Pathway classifications of differentially expressed genes according to the KEGG results (left, WF vs. WM; right, GF vs. GM).

Multiple comparisons were conducted between the gonads of the WF vs. WM and GF vs. GM groups to evaluate the differential expression of genes between the ovary and testis. Overall, a total of 23,127 genes were identified from the 12 flounder gonadal transcriptomes in this study, and a large number of them showed sex-biased expression (fold change > 2 and adjusted *p*-value ≤ 0.05). The DEG numbers in different groups are shown in Figure 7A. There were 12,402 and 11,897 genes showing different expression levels between the WF vs. WM and GF vs. GM, respectively. Compared with WF, 6,838 upregulated and 5,564 downregulated unigenes were identified in WM, while 6,446 upregulated and 5,451 downregulated unigenes were identified in GM compared to GF (Figure 7A). Principal component analysis (PCA) plot and heatmap both showed that differences in gene expression were much more pronounced between the ovary and testis than between the wildtype and gynogenetic gonads (Figures 7B,C). Compared with the wildtype ovary, 300 upregulated and 927 downregulated unigenes were identified in the gynogenetic ovary, while compared with the wildtype testis, 288 upregulated and 212 downregulated unigenes were identified in the gynogenetic testis.

As expected, *cyp19a1a* [aromatase converting testosterone (T) into E2] expression was higher in the ovary. However, 11β-hydroxylase (*cyp11b*) and 11β-hydroxysteroid dehydrogenase 2 (*hsd11b2*), which convert T into 11-KT, were not detected in either the ovary or testis. In addition, some genes involved in sex steroid synthesis mostly showed higher expression levels in the testis than in the ovary, such as steroidogenic acute regulatory protein gene (*star1*), cytochrome P450c17 (*cyp17a1*), and 3β-hydroxy-Δ5-C27-steroid oxidoreductase (*hsd3b7*), while the 17β-hydroxysteroid dehydrogenase 1 gene (*hsd17b1)* showed female-biased expression.

The WF vs. WM and the GF vs. GM comparisons revealed 14 and 13 significantly different KEGG pathways, respectively (Figure 7D and Supplementary Table S5). The results showed that DEGs identified in the WF vs. WM comparison were related to the pathways of “Cellular senescence,” “Phenylalanine metabolism,” “Glycosylphosphatidylinositol (GPI)-anchor biosynthesis,” “Small cell lung cancer,” “Sphingolipid metabolism,” “Arachidonic acid metabolism,” “Ribosome biogenesis in eukaryotes,” “Terpenoid backbone biosynthesis,” “GnRH signaling pathway,” “Fatty acid biosynthesis,” “Protein processing in endoplasmic reticulum,” “Glycosaminoglycan biosynthesis – chondroitin sulfate/dermatan sulfate,” “Antifolate resistance,” and “Thyroid hormone signaling pathway,” while the DEGs identified in the GF vs. GM comparison were related to “Ribosome biogenesis in eukaryotes,” “Phenylalanine metabolism,” “Thyroid cancer,” “Primary bile acid biosynthesis,” “Sphingolipid metabolism,” “Arginine and proline metabolism,” “Terpenoid backbone biosynthesis,” “Arachidonic acid metabolism,” “Glycerophospholipid metabolism,” “Glycerolipid metabolism,” “Limonene and pinene degradation,” “Other types of *O*-glycan biosynthesis,” and the “PPAR signaling pathway.”

## Discussion

### RNA Editing in the Flounder Ovary and Testis

RNA editing can increase transcriptome diversity and flexibility without mutations occurring at the DNA level (Hwang et al., 2016). It can affect the structure and coding of RNA and generate proteomic diversity (Koslowsky, 2004). After the identification of the RNA editing in trypanosomes in 1986, the RNA editome was well characterized in mammals such as humans (Peng et al., 2012), mice (Danecek et al., 2012), bovines (Bakhtiarizadeh et al., 2018), and porcines (Zhang et al., 2019). However, no related reports have been published for fish species with the exception of the report by Guryev et al. (2006) describing the presence of C-to-U and A-to-I RNA editing events in zebrafish. In this study, we systematically detected and characterized the RNA editome in the gonads of the flounder based on RNA and corresponding DNA sequencing data. The results showed that the canonical A-to-I RNA editing event was the most common type of editing in the flounder, which was consistent with previous studies in mammalian species (Hwang et al., 2016; Bakhtiarizadeh et al., 2018). However, most of the editing sites in the flounder gonads targeted non-coding regions (3′UTRs), which differs from findings in humans (intronic) (Nishikura, 2016) and porcines (intergenic) (Zhang et al., 2019). Compared to mammals, the ratio of editing sites in coding regions is higher in the flounder. The large number of editing sites occurring in introns and 3′UTRs of the flounder gonads indicated that RNA editing in fish may play fundamental roles in the regulation of splicing and miRNA regulation, respectively (Hwang et al., 2016).

Notably, our results also showed that the number of editing sites was different between the ovary and testis. Compared to the testis, the lower number of editing sites identified in the ovary suggested lower RNA editing activity. Most of the genes harboring RNA editing sites were more highly expressed in the testis than in the ovary. Thus, RNA editing may regulate gene expression, but the regulatory mechanism will be subject to further study. Annotation of the edited genes in the flounder gonads revealed that some of the genes were associated with gonadal development and gametogenesis. For instance, *cullin3*, a component of the Cullin-3-based E3 ubiquitin ligase complex, is necessary for caspase activation. Mutations in this gene reduce the effect of caspase activation and block apoptosis during spermatid individualization (Richburg et al., 2014). *Srpk2* is highly expressed in mouse testis and might participate in precursor mRNA splicing during mouse spermiogenesis (Yang et al., 2015). *Dazap2* interacts with the germ-cell-specific RNA-binding proteins Daz and Dazl1 (Yen, 2004).

Similar to what is observed in mammals, three independent genes of the ADAR family were identified in the flounder transcriptome: *adar* (*adar1*), *adarb1* (*adar2*), and *adarb2* (*adar3*). According to studies in mice, ADAR enzymes are essential for normal life and development, and knockout or homozygous null mutations in ADAR genes lead to lethality (Higuchi et al., 2000; Wang et al., 2000). In the flounder, *adar1* showed the highest expression level among these three genes, while *adar3* showed a very low expression level in the gonads. Only *adar1* and *adar2* have been shown to be enzymatically active (Nishikura, 2016), while *adar3* is exclusively expressed in the brain and can inhibit RNA editing by competitively binding double-stranded RNA (Picardi et al., 2015; Oakes et al., 2017). Our results also proved that the distribution of A-to-G sites was significantly and positively correlated with the expression of *adar1*, suggesting that editing enzyme expression may play an essential role in regulating tissue-specific editing levels.

### M6A Profiles of the Flounder Gonads

M6A represents the most prevalent epigenetic modification of RNAs. Many m6A profiling studies have been performed in mammals, avians, and plants, but no related reports have been published for fish except for zebrafish (Zhang et al., 2017). A study in zebrafish revealed conserved features of the m6A methylome and preferential distribution of m6A peaks near stop codons with a consensus RRACH motif. By using the MeRIP-seq method, we produced the first transcriptome-wide m6A modification profile and detected m6A methylation sites in the flounder gonadal transcriptome. There were approximately 1.7 m6A peaks per transcript, which was similar to the situation in zebrafish (1.7) (Zhang et al., 2017) but slightly higher than the number found in mammals (1.5) (Dominissini et al., 2012). M6A modification can regulate gene function by controlling RNA splicing, nuclear export, and RNA degradation and translation (Frye et al., 2018), and is profoundly implicated in biological processes in animal gonads. Studies show that deficiency of the *alkbh5* demethylase leads to aberrant spermatogenesis and apoptosis with impaired fertility in the testis (Zheng et al., 2013), while *ythdf2* is required for the generation of the maternal transcriptome during oocyte maturation (Ivanova et al., 2017), and *ythdc1* is essential for the production of spermatogonia in males and oocyte maturation in females (Kasowitz et al., 2018). Furthermore, the simultaneous deletion of *mettl14* and *mettl3* in advanced mouse germ cells results in a decrease in sperm motility and an increase in the abnormal sperm ratio (Lin et al., 2017). In fish, the understanding of the function of m6A is still limited. It has been proven that zebrafish *mettl3* is essential for sperm maturation and motility and can regulate 11-KT and E2 levels (Xia et al., 2018). In our study, the expression of the “writers” *mettl14* and *cbll1* was found to be more highly abundant in the ovary than in the testis, while the “erasers” *fto* and *alkbh5* and the “readers” *ythdf1* and *ythdc2*, which increase the translation of methylated mRNA, were expressed at especially high levels in the testis.

Several genes that have been reported as playing important roles or participating in sex determination/differentiation or gametogenesis showed differential m6A RNA methylation in the flounder ovary and testis. For example, *dmrt1*, *amh*, *dazl*, and Sox protein genes (*sox4, sox8*, *sox9*, and *sox11*) are important for the regulation of testis development and spermatogenesis (Barrionuevo and Scherer, 2010; Pfennig et al., 2015; Webster et al., 2017). *Spag5*, -*8*, -*17*, and *spata7* were significantly more highly expressed in the testis than in the ovary, and the expression difference might be associated with spermatogenesis (Yang et al., 2016). The zona pellucida (ZP) is an extracellular glycoproteinaceous matrix shell that is involved in oocyte and gamete development, and the expression levels of *zp* gene mRNAs were found to be significantly increased during oogenesis in fish, especially at the previtellogenic stage, when they showed a higher expression level than that at the undeveloped stage (Zeng and Gong, 2002). *Zp3* showed higher expression levels in the flounder ovary compared to the testis, and previous studies showed that this gene represents a major class of female-specific molecule with a role in reproduction (Liu et al., 2006). Although differential m6A modification of these genes between the ovary and testis was detected, the functions of these genes and their regulation by m6A modification are still unknown and should be further studied.

The DEGs with differential m6A RNA methylation were enriched in several canonical signaling pathways. These included pathways involved in gonadal differentiation and development in fish and other vertebrates, such as the TGF-beta signaling pathway, Wnt signaling pathway, ovarian steroidogenesis pathway, and FoxO signaling pathway. Among these pathways, the FoxO signaling pathway was enriched in both the WF vs. WM and GF vs. GM comparisons. FoxO proteins of different subclasses are transcription factors that play important roles in cell cycle arrest, apoptosis, and stress responses *in vitro* (Taisuke et al., 2004). Biochemical and genetic studies in mice have implicated the Pten/Pi3k/Akt/FoxO3 pathway as a major signaling pathway involved in the regulation of dormancy and initial follicular activation in the ovary (Lee and Chang, 2019). In the current study, the mRNAs encoding *pten, pi3k*, and *foxo3* were all modified by m6A methylation. Intriguingly, they showed opposite m6A distribution patterns between the ovary and testis. Our results also included pathways whose roles have not been previously investigated in depth in relation to fish sex differentiation and gonadal development, such as the PPAR and RNA degradation pathways. A previous study in mammals indicated that PPAR is capable of regulating the function of the reproductive organs by modulating the expression of related steroidogenic enzymes through Star (Kowalewski et al., 2009) and PPAR-γ can regulate progesterone production in ovarian granulosa cells by coactivation with *sf1* and *lhxr1* (Yazawa et al., 2010). PPAR and the key signaling molecule RXRG in this pathway can promote the differentiation of chicken embryonic stem cells into primordial germ cells (Cheng et al., 2018). In this study, the m6A RNA methylation levels of several critical genes in this pathway, including *sirt1* and *RXR*, were found to be different in the ovary and testis, but its function remains unclear in the gonads. The GO analysis showed that the cilia-related genes were significantly enriched. Primary cilium was identified as a key coordinator of signaling during organogenesis, and it had been shown that the primary cilium participated in receiving signals from Notch, PDGFRα, FGF, Wnt, and Hedgehog pathways (Wainwright et al., 2014). Primary cilia-related genes are present in different cell types of gonads and may be important for the differentiation of gonad cells and sex differentiation of gonads in mammals (Piprek et al., 2019). However, the role of primary cilium in the sex determination and gonadal differentiation in fish is completely unknown. The possible direct and indirect effects mediated by m6A modification remain to be further elucidated.

### The Relationship Between m6A and A-to-I Editing in the Flounder Gonads

Among the different types of RNA modifications, m6A and A-to-I editing are two of the most abundant. Although both modifications occur on A bases, their catalytic mechanisms are distinct. A-to-I conversion is catalyzed by ADARs that preferentially bind to double-stranded RNA substrates (Nishikura, 2016), which are dependent on the formation of RNA secondary structures (Bahn et al., 2015). In contrast, m6A RNA methylomes exhibit enrichment of the RRACH motif at m6A sites. Thus, the different sequences and structural features of the A-to-I and m6A modifications suggest that these two chemical modifications are unlikely to modify the same A bases. Xiang et al. (2018) demonstrated a global A-to-I difference between m6A-positive and m6A-negative RNA populations in mammals and indicated that there was a negative correlation between m6A and A-to-I modifications (Xiang et al., 2018). In this study, 125 genes were found to be modified by both the m6A and A-to-I editing, 30 of which showed significantly different expression levels between the ovary and testis. Most of the positions of the m6A modifications and RNA editing were not coincident, which was in accordance with previous research results (Xiang et al., 2018). However, the regulatory relationship between the A-to-I and m6A modifications is unclear in the flounder and requires further study.

The teleost gonadal development is a dynamic process with various phases that are similar in different species. Testicular stage is generally based on the relative proportions of spermatocytes, spermatids, and spermatozoa (Blazer, 2002). Histological analysis suggested the testes used in this study were at stage IV to V. At stage IV, the primary spermatocytes, secondary spermatocytes, and spermatids were arranged in tubule seminiferous. Some of the spermatids developed into spermatozoa. While at stage V, the mature spermatozoa predominated in the testis and the number of spermatocytes was obviously less than that at stage IV. Thus, the sampled testes were at spermiogenesis stage with an increasing proportion of spermatozoa. Numerous well documented sex-related genes involved in gonadal development and gametogenesis were differently modified between the ovary and testis in this study. Among the candidate genes, a subset of well-established genes involved in spermatogenesis (e.g., *dazl, dazap2*, *amh*, *gadd45g*, *srpk2*, *Spag5*, *-8*, *-17*, and *spata7*) were detected. For instance, *dazl* is a master translational regulator essential for spermatogenesis (Li et al., 2019). *Amh* is expressed in the Sertoli cells that surrounded the germ cells and involved in the maintenance of the flounder testis (Wang et al., 2020). *Gadd45g* is necessary for activation of the male sexual pathway in mice and its absence leads to sex reversal (Johnen et al., 2013). In contrast, the developmental stage of the ovary is classified based on ogenesis process, such as oocyte size, presence of follicular layer, and number of nucleoli. The ovaries in this study were at stage II with a large number of phase II oocytes which were bigger than oogonia. Correspondingly, several genes related to ovarian or oocyte development (e.g., *bmp15*, *foxo3*, and *nr0b1*) were identified. The *bmp15*, an oocyte-expressed signaling molecule, is required for maintenance of the female sexual fate in zebrafish (Dranow et al., 2016). The *foxo3* is a critical transcriptional regulator of fish ovarian development by regulating the ovarian germ cells and follicular cells (Liu et al., 2016). *Nr0b1*, also called *dax1*, mainly locates in fish primary oocytes and previtellogenic oocyte cells, and its mutation in zebrafish causes female-to-male sex reversal (Chen et al., 2016). In this study, we obtained a global view of the RNA modification differences between male and female fish gonads. Nevertheless, the gonadal development is a dynamic process with different gene expression patterns at different developmental stages. As specific developmental stage samples were used, this study could not elucidate the dynamics of gene expression and RNA modifications over the course of gonadal development. Thus, the dynamic changes of the RNA modifications and gene expression at all gonadal development stages should be analyzed in the near future. Moreover, it should be noted that the RNA modification patterns of gonads may differ depending on the cell types. As the entire gonads were used in the present study, the RNA modifications in this study represent the combination of the different cell types. Further study focusing on single-cell analysis would increase our knowledge of gonadal development and sexual plasticity in fish.

RNA editing and m6A have been recognized as targets for drug discovery, and some drugs for inhibiting RNA editing enzymes or m6A modification regulators were discovered recently (Christofi and Zaravinos, 2019; Ianniello et al., 2019). Our study showed the levels of the RNA editing and m6A were different in the flounder ovary and testis, and some sex-related genes (e.g., *dmrt1, amh*, and *wt1*) may be regulated by RNA modifications. Therefore, these RNA modification regulators and sex-related genes provide new potential targets for therapeutic products in fish sex control. However, the mechanisms of sex determination in fish are extremely complex, and identifying and understanding biological functions of RNA modification in fish sex determination and differentiation are important for designing reasonable chemical/drug.

Despite the substantial results have obtained in this study, some restrictions due to sequencing limitations remained. MeRIP-seq is the most common method to elucidate global mRNA m6A sites, but this approach could not accurately reflect the positions of m6A residues (Liu et al., 2013). The methylated regions are detected as peaks of approximately 100–200 base pairs that contain multiple DRACH motifs in transcript relative to input RNA. Moreover, antibodies for m6A can also detect a second base modification, *N6*,2′-*O*-dimethyladenosine (m6Am), which has been found at a lower abundance than m6A (Mauer and Jaffrey, 2018). Another common method for transcriptome-wide studies of m6A was the m6A individual-nucleotide-resolution cross-linking and immunoprecipitation (miCLIP), which is not influenced by peak shapes and not restricted to DRACH motifs (Linder et al., 2015). However, MeRIP-seq is still more often used than miCLIP, as it follows a simpler protocol and requires less starting RNA (McIntyre et al., 2020). Next-generation sequencing (NGS) technologies enable genome-wide identification of RNA-editing sites, and RNA editing studies are widely implemented by comparing matched high-throughput RNA sequencing and DNA sequencing data currently (Wang et al., 2016; Zhang et al., 2019). Currently, many bioinformatics tools can identify RNA-editing sites from RNA-seq data, but the accurate identification of the RNA editing sites may suffer from methodical artifacts such as reverse transcription errors, sequencing errors, alignment errors, and single nucleotide polymorphisms (SNP) (Wang et al., 2016; Guo et al., 2017). Thus, individual-nucleotide-resolution of m6A modification in the flounder gonads and the verification of identified RNA editing sites should be carried out in the subsequent study.

### Gynogenetic Flounder Is an Appropriate Model for Studying Epigenetic Factors Involved in Fish Sex Determination and Differentiation

Gynogenesis is a form of genetically all-female reproduction in which the male pronucleus does not fuse with the female pronucleus in gynogenetic zygotes (Neaves and Baumann, 2011). The flounder exhibits an XX (female)/XY (male) sex determination system, and all gynogenetic female flounders should exhibit an XX genotype. However, the sex determination and gonadal development of the flounder are determined by genetic factors but are also affected by environmental factors, and even gynogenetic individuals can produce a male phenotype. Thus, the gynogenetic flounder possess same genome is able to produce two distinct sexual phenotypes in response to environmental cues with mechanisms not involving a genomic change. Epigenetic modifications provide an interface to integrate genetic factors and environmental signals during sex determination. Epigenetic factors such as DNA modifications have been reported to participate in the flounder sex differentiation. Our previous studies showed that the *cyp19a* promoter exhibited dimorphic methylation levels between the flounder testis and ovary, which were inversely correlated with its expression levels (Wen et al., 2014; Fan et al., 2017). To date, the epigenetic studies in fish sex determination mainly focus on heritable modifications of DNA, histones, and chromatin structure (Ortega-Recalde et al., 2020). RNA modifications such as RNA editing and m6A RNA methylation may shed new light on fish sex determination and development. However, as epitranscriptomic biomarkers, the roles of RNA editing and m6A modifications in the regulation of sex differentiation remain largely unknown. XX female and male fish could provide an appropriate model for studying this topic. Our previous study demonstrated that gene expression in gynogenetic diploids was similar to that in common diploids (Fan et al., 2016), which is consistent with the results of this study. The present study also showed that the identified RNA modifications were similar in the wildtype and gynogenetic gonads. For instance, the RNA editing sites and levels were almost the same, and there was no significant difference in the position or level of the m6A peaks between the wildtype and gynogenetic gonads.

Up till now, the studies of RNA modifications in sex determination were carried out in only a few model animals, such as *Drosophila* and zebrafish (Haussmann et al., 2016; Lence et al., 2016; Xia et al., 2018). In *Drosophila*, m6A can regulate sex by facilitating the alternative splicing of the key sex determination factor *sxl*. In zebrafish, knockout of the m6A methyltransferase *mettl3* led to a defect in fertility as well as sex differentiation. These studies all focused on a specific modification regulator, and used the mutants to study its functions. The relatively long generation interval of the flounder causes a great deal of discomfort on such function researches. Nevertheless, compared to the zebrafish with a complex polygenic sex determining system, the gynogenetic flounder exhibits an XX female genotype with different phenotypic sexes. Sex differentiation of the flounder may be regulated by mechanisms not only involving genomic changes, but also involving epigenetic factors. Thus, the gynogenetic flounder probably provides a better model for addressing the epigenetic modification on fish sex determination issues. Moreover, the flounder is an aquaculture fish that is yet to reach industrial scale production, and elucidation of it sex differentiation is also a key area of applied research.

In conclusion, we profiled the RNA editing and m6A methylomes of the flounder ovary and testis. The results indicated that most A-to-I sites were located in the 3′UTRs of the mRNAs, and the number of the editing sites in the testis was higher than that in the ovary. The flounder gonadal transcriptome was extensively methylated with the m6A modifications, and the different peaks were mostly located in CDs and 3′UTRs of the mRNAs. The RNA editing and m6A modifications of RNA may be involved in the regulation of gonadal development and gametogenesis in the flounder gonads. The high-throughput epitranscriptome data in this study provide a foundation for understanding these critical RNA modifications in fish and expand our knowledge of the function of RNA modifications. Further researches using gonads at all developmental stages and during gonadal differentiation period, even using single cell type of gonads, are needed to shed light on the roles of RNA editing and m6A modifications in sexual plasticity and gonadal development of fish.

## Materials and Methods

### Fish and Sample Collection

The meiogynogenetic flounders were obtained through artificial induction as previously reported (You et al., 2001). Briefly, sperm were irradiated under ultraviolet light and then used to be fertilized with eggs. Five minutes after fertilization, the fertilized eggs were treated under cold shock by being immersed in 0°C seawater for 45 min, after which the eggs were incubated in normal seawater, and fertilized eggs that were not subjected cold shock were used as the haploid control group to confirm meiogynognesis induction. The fertilized eggs obtained with non-irradiated sperm and ordinary eggs were reared under same conditions as the control individuals (wildtype). The sexes of the meiogynogenetic and control fishes were identified by morphological observation of the gonads at approximately 1.5 years of age, and the developmental stages of the gonads were detected by using the histological method described as Sun et al. (2009). Gonadal and muscle tissues of three males (wildtype males, WM, 29.82 ± 0.86 cm total length, TL; 322.96 ± 47.36 g body weight, BW) and three females (wildtype females, WF, 31.95 ± 1.51 cm TL; 399.17 ± 50.84 g BW) from the flounders in the control group, and three males (gynogenetic males, GM, 29.71 ± 0.30 cm TL; 337.83 ± 37.32 g BW) and three females (gynogenetic females, GF, 31.84 ± 0.71 cm TL; 417.20 ± 31.63 g BW) from the flounders in the induced meiogynogenetic diploid group were collected. Gonadal and muscle samples were retrieved from the fish after they were anaesthetized with MS-222 (Sigma, United States). Each gonadal sample was divided into two halves. One half was fixed in Davison’s fixative solution for histological analysis, and the other half was immediately stored in liquid nitrogen for RNA isolation. The muscle samples were frozen immediately and then stored at −80°C for DNA extraction.

### Nucleic Acid Isolation

Genomic DNA was extracted from the muscle samples by using the standard phenol-chloroform protocol. Only DNA samples with OD 260/280 ratios of 1.8–2.0 and total contents greater than 1.5 μg were used in the subsequent steps. Total RNA was isolated from the gonads using TRIzol reagent (Invitrogen, United States) according to the manufacturer’s instructions. The quality and quantity of the RNA were analyzed with Nanodrop 2000 (Thermo Fisher Scientific, United States) and Agilent 2100 Bioanalyzer (Agilent Technologies, United States) instruments, respectively. Testis RNA samples with RNA integrity number (RIN) scores higher than seven were used in this study. As in other fishes, the flounder ovary samples showed a strong peak of low-molecular-weight RNA, which masked the 18S and 28S rRNA peaks used for calculating the RIN. Therefore, the RIN score could not be used to measure RNA integrity in the ovary.

### Transcriptome Sequencing

Three micrograms of total RNA per gonad were used for the subsequent generation of RNA-seq libraries. In total, 12 sequencing libraries were generated using mRNA purified from total RNA using TruSeq RNA Sample Preparation Kit (Illumina, San Diego, CA, United States) following the manufacturer’s protocol. Each library preparation was sequenced on an Illumina HiSeq platform (Shanghai Personal Biotechnology Co., Ltd., Shanghai, China), and 150 bp paired-end reads were generated. The sequencing data were filtered with Cutadapt v2.7^1^ (Martin, 2011). RNA-seq reads were mapped to the olive flounder genome using HISAT2^2^ (Kim et al., 2015) with the default mismatch being no more than 2. HTSeq^3^ (Anders et al., 2015) was used to compare the Read Count values on each gene as the original expression of the gene, and then reads per kilobase per million (FPKM) was used to standardize the expression. DESeq v1.39.0 (Anders and Huber, 2010) was used to analyze the genes of different expression. Differentially expressed genes (DEGs) were selected on the basis of a log2 (fold change) > 1 or log2 (fold change) < −1 and a false discovery rate (FDR) < 0.05. Bi-directional clustering analysis of all different genes of samples was performed by R language Pheatmap v1.0.12 software package. The topGO v2.40.0 was used to map all the genes to terms in the Gene Ontology database and calculate the numbers of differentially enriched genes in each term.

### Whole-Genome Sequencing

Muscle DNA from the 12 fishes was used to construct 12 DNA libraries. These libraries were generated using a TruSeq Nano DNA HT Sample Preparation Kit (Illumina, San Diego, CA, United States) according to the manufacturer’s recommendations. Then, each library was sequenced on an Illumina HiSeq platform (Shanghai Personal Biotechnology Co., Ltd., Shanghai, China), and 150 bp paired-end reads were generated for further analysis (10 × depth). The quality control of the raw reads was conducted with FastQC software v0.11.2^4^. The raw data were trimmed and filtered with AdapterRemoval and trimmomatic v. 0.36 (Bolger et al., 2014) and then were aligned to the reference genome. The reference flounder genome was downloaded from Ensembl (GCF_001970005.1_Flounder_ref_guided_V1.0).

### RNA Editing Detection

RNA editing sites were detected by direct comparison between the RNA-seq data from the flounder gonads and the corresponding genomic data using RES-Scanner. All thresholds used for the identification of RNA editing sites were the default parameters of RES-Scanner (Wang et al., 2016). The low frequency variant detection parameters were set to 5%, and the minimum coverage, minimum count, and minimum frequency were set to 10, 2, and 5%, respectively. A site was considered to be a potential editing site when it was detected in at least two individuals. The statistical significance of the differences between the samples in terms of the editing ratio was assessed with Student’s unpaired *t*-test was implemented in SPSS16.0.

### High-Throughput m6A and Input RNA Sequencing

Poly (A) RNA was purified from 200 μg total RNA using Dynabeads Oligo (dT) 25-61005 (Thermo Fisher, CA, United States) with two rounds of purification. Following purification, the poly (A) RNA was fragmented into small pieces using Magnesium RNA Fragmentation Module (NEB, cat.e6150, United States) for 7 min under 86°C. Then the cleaved RNA fragments were incubated for 2 h at 4°C with m6A-specific antibody (No. 202003, Synaptic Systems, Germany) in IP buffer (50 mM Tris-HCl, 750 mM NaCl, and 0.5% Igepal CA-630). The IP RNA was reverse-transcribed to create the cDNA by SuperScript^TM^ II Reverse Transcriptase (Invitrogen, cat. 1896649, United States), which were next used to synthesis U-labeled second-stranded DNAs with *E. coli* DNA polymerase I (NEB, cat.m0209, United States), RNase H (NEB, cat.m0297, United States), and dUTP Solution (Thermo Fisher, cat.R0133, United States). An A-base was then added to the blunt ends of each strand, preparing them for ligation to the indexed adapters. Each adapter contained a T-base overhang for ligating the adapter to the A-tailed fragmented DNA. Single- or dual-index adapters were ligated to the fragments, and size selection was performed with AMPureXP beads. After the heat-labile UDG enzyme (NEB, cat.m0280, United States) treatment of the U-labeled second-stranded DNAs, the ligated products were amplified with PCR with the following conditions: initial denaturation at 95°C for 3 min; 8 cycles of denaturation at 98°C for 15 s, annealing at 60°C for 15 s, and extension at 72°C for 30 s; and then final extension at 72°C for 5 min. The average insert size for the paired-end libraries was ∼100 ± 50 bp. At last, we performed the 2 × 150 bp paired-end sequencing (PE150) on an illumina Novaseq^TM^6000 (LC-Bio Technology CO., Ltd., Hangzhou, China) following the vendor’s recommended protocol.

The fastp software^5^ was used to remove the reads that contained adaptor contamination, low quality bases, and undetermined bases with default parameter. Then sequence quality of IP and Input samples was also verified using fastp. We used HISAT2 to map reads to the genome of the flounder with default parameters. The mapped reads of IP and input libraries were analyzed with R package exomePeak^6^ (Meng et al., 2014), which identified m6A peaks with bed or bigwig format that were adapted for visualization on the IGV software^7^. MEME^8^ (Bailey et al., 2009) and HOMER^9^ (Heinz et al., 2010) were used for *de novo* motif identification, followed by localization of the motif with respect to peak summit. Called peaks were annotated by intersection with gene architecture using R package ChIPseeker^10^ (Yu et al., 2015). Then StringTie^11^ (Pertea et al., 2015) was used to perform expression level for all mRNAs from input libraries by calculating FPKM. The differentially expressed mRNAs were selected with log2 (fold change) > 1 or log2 (fold change) < −1 and *p-*value < 0.05 by R package edgeR^12^ (Robinson et al., 2010).

### Validation of Transcriptomic Data

Real-time quantitative polymerase chain reaction (qPCR) was performed to verify the expression profiles obtained from the transcriptomic data. The RNA used for qPCR was the same RNA used for RNA-seq analysis. Ten m6A methyltransferase genes (*mettl3, mettl14, wtap, fto, alkbh5, ythdc1, ythdc2, ythdf1, ythdf2*, and *ythdc3*) and three *adar* genes (*adar1, adar2*, and *adar3*) were chosen for analysis. The primers used in this study were designed based on sequences from the transcriptome using Primer Premier 6.0 (PREMIER Biosoft International, United States) and are listed in Supplementary Table S6. Three replicates were performed for each gene in each individual. The relative quantification result for each target gene was expressed as the fold variation over the reference genes (*ef1*α and β-*actin*) (Zhong et al., 2008; Zheng and Sun, 2011) as calculated with the 2^–ΔΔCt^ comparative Ct method. PCR specificity was assessed through melting curve analysis. All data are expressed as the means ± SE (standard error). *Post hoc* Duncan’s multiple range tests were used to determine the differences between groups using SPSS 16.0. Significance was set at *p* < 0.05.

## Data Availability Statement

The datasets generated for this study can be found in the NCBI Sequence Read Archive BioProject PRJNA639001, PRJNA638896.

## Ethics Statement

The study was conducted in accordance with the guidelines and regulations established under the Chinese Government Principles for the Utilization and Care of Animals  Used in Testing, Research, and Training. And the experiments were conducted strictly with the ethical standards of the Guidelines of Regulations for the Administration of Affairs Concerning Experimental Animals documented by the State Science and Technology Commission of Shandong Province. All the researchers who performed the experiments have been appropriately trained. The animal work and animal protocols were approved by the Institute of Oceanology, Chinese Academy of Sciences.

## Author Contributions

FY designed the experiments and supervised the study. LW and ZW designed and participated the sample collection. LW, CZ, and SL conducted the experiments. LW, CZ, YZ, and YL performed the data analysis. LW and FY wrote and edited the manuscript. All the authors read and approved the final manuscript.

## Conflict of Interest

The authors declare that the research was conducted in the absence of any commercial or financial relationships that could be construed as a potential conflict of interest.

## Footnotes

1 http://code.google.com/p/cutadapt/

2 http://daehwankimlab.github.io/hisat2

3 https://pypi.org/project/HTSeq/

4 https://www.bioinformatics.babraham.ac.uk/projects/fastqc

5 https://github.com/OpenGene/fastp

6 https://bioconductor.org/packages/exomePeak

7 http://www.igv.org

8 http://meme-suite.org

9 http://homer.ucsd.edu/homer/motif

10 https://bioconductor.org/packages/ChIPseeker

11 https://ccb.jhu.edu/software/stringtie

12 https://bioconductor.org/packages/edgeR

## Supplementary Material

TABLE S1 List of the identified RNA editing sites in the flounder gonads.

TABLE S2 Statistical significance analysis for comparing the editing ratio in different gonadal tissues.

TABLE S3 GO enrichment analysis of DGEs with differential RNA methylation level.

TABLE S4 Pathway enrichment analysis of DGEs with differential RNA methylation level.

TABLE S5 Pathway enrichment analysis of DGEs between the flounder ovary and testis.

TABLE S6 Primers used in real-time quantitative polymerase chain reactions with their melting temperature.

## References

[B1] Alarcón C. R. Hyeseung L. Hani G. Nils H. Tavazoie S. F. (2015). N6-methyladenosine marks primary microRNAs for processing. *Nature* 519 482–485. 10.1038/nature14281 25799998 PMC4475635

[B2] Anders S. Huber W. (2010). Differential expression analysis for sequence count data. *Genome Biol.* 11:R106. 10.1186/gb-2010-11-10-r106 PMC3218662 20979621

[B3] Anders S. Pyl P. T. Huber W. (2015). HTSeq-a Python framework to work with high-throughput sequencing data. *Bioinformatics* 31 166–169. 10.1093/bioinformatics/btu638 25260700 PMC4287950

[B4] Bahn J. H. Ahn J. Lin X. Zhang Q. Lee J.-H. Civelek M. (2015). Genomic analysis of ADAR1 binding and its involvement in multiple RNA processing pathways. *Nat. Commun.* 6:6355. 10.1038/ncomms7355 PMC4355961 25751603

[B5] Bailey T. L. Boden M. Buske F. A. Frith M. Grant C. E. Clementi L. (2009). MEME SUITE: tools for motif discovery and searching. *Nucleic Acids Res.* 37 W202–W208. 19458158 10.1093/nar/gkp335 PMC2703892

[B6] Bakhtiarizadeh M. R. Salehi A. Rivera R. M. (2018). Genome-wide identification and analysis of A-to-I RNA editing events in bovine by transcriptome sequencing. *PLoS One* 13:e0193316. 10.1371/journal.pone.0193316 29470549 PMC5823453

[B7] Barrionuevo F. Scherer G. (2010). SOX E genes: SOX9 and SOX8 in mammalian testis development. *Int. J. Biochem. Cell Biol.* 42 433–436. 10.1016/j.biocel.2009.07.015 19647095

[B8] Batista P. J. Benoit M. Jinkai W. Kun Q. Jiajing Z. Lingjie L. (2014). m(6)A RNA modification controls cell fate transition in mammalian embryonic stem cells. *Cell Stem Cell* 15 707–719. 10.1016/j.stem.2014.09.019 25456834 PMC4278749

[B9] Bazak L. Haviv A. Barak M. Jacob-Hirsch J. Deng P. Zhang R. (2014). A-to-I RNA editing occurs at over a hundred million genomic sites located in a majority of human genes. *Genome Res.* 24 365–376. 10.1101/gr.164749.113 24347612 PMC3941102

[B10] Blazer V. S. (2002). Histopathological assessment of gonadal tissue in wild fishes. *Fish Physiol. Biochem.* 26 85–101.

[B11] Bolger A. M. Lohse M. Usadel B. (2014). Trimmomatic: a flexible trimmer for Illumina sequence data. *Bioinformatics* 30 2114–2120. 10.1093/bioinformatics/btu170 24695404 PMC4103590

[B12] Capel B. (2017). Vertebrate sex determination: evolutionary plasticity of a fundamental switch. *Nat. Rev. Genet.* 18:675. 10.1038/nrg.2017.60 28804140

[B13] Chen S. Zhang H. Wang F. Zhang W. Peng G. (2016). Nr0b1 (DAX1) mutation in zebrafish causes female-to-male sex reversal through abnormal gonadal proliferation and differentiation. *Mol. Cell. Endocrinol.* 433 105–116. 10.1016/j.mce.2016.06.005 27267667

[B14] Cheng S. Wang M. Wang Y. Zhang C. Wang Y. Song J. (2018). RXRG associated in PPAR signal regulated the differentiation of primordial germ cell. *J. Cell. Biochem.* 119 6926–6934. 10.1002/jcb.26891 29738084

[B15] Christofi T. Zaravinos A. (2019). RNA editing in the forefront of epitranscriptomics and human health. *J. Transl. Med.* 17:319. 10.1186/s12967-019-2071-4 PMC6757416 31547885

[B16] Danecek P. Nellåker C. Mcintyre R. E. Buendia-Buendia J. E. Bumpstead S. Ponting C. P. (2012). High levels of RNA-editing site conservation amongst 15 laboratory mouse strains. *Genome Biol.* 13:26. 10.1186/gb-2012-13-4-r26 PMC3446300 22524474

[B17] Dominissini D. Moshitch-Moshkovitz S. Schwartz S. Salmon-Divon M. Ungar L. Osenberg S. (2012). Topology of the human and mouse m6A RNA methylomes revealed by m6A-seq. *Nature* 485 201. 10.1038/nature11112 22575960

[B18] Dranow D. B. Hu K. Bird A. M. Lawry S. T. Adams M. T. Sanchez A. (2016). Bmp15 is an oocyte-produced signal required for maintenance of the adult female sexual phenotype in zebrafish. *PLoS Genet.* 12:e1006323. 10.1371/journal.pgen.1006323 27642754 PMC5028036

[B19] Du K. Zhang L. Lee T. Sun T. (2019). m6A RNA methylation controls neural development and is involved in human diseases. *Mol. Neurobiol.* 56 1596–1606. 10.1007/s12035-018-1138-1 29909453

[B20] Fan Y. Zhang C. Zhu G. (2019). Profiling of RNA N6-methyladenosine methylation during follicle selection in chicken ovary. *Poultry Sci.* 98 6117–6124. 10.3382/ps/pez277 31189182

[B21] Fan Z. Wu Z. Wang L. Zou Y. Zhang P. You F. (2016). Characterization of embryo transcriptome of gynogenetic olive flounder *Paralichthys olivaceus*. *Mar. Biotechnol.* 18 545–553. 10.1007/s10126-016-9716-6 27638397

[B22] Fan Z. Zou Y. Jiao S. Tan X. Wu Z. Liang D. (2017). Significant association of cyp19a promoter methylation with environmental factors and gonadal differentiation in olive flounder *Paralichthys olivaceus*. *Comp. Biochem. Physiol. A Mol. Integr. Physiol.* 208 70–79. 10.1016/j.cbpa.2017.02.017 28219743

[B23] Farajollahi S. Maas S. (2010). Molecular diversity through RNA editing: a balancing act. *Trends Genet.* 26 221–230. 10.1016/j.tig.2010.02.001 20395010 PMC2865426

[B24] Frye M. Harada B. T. Behm M. He C. (2018). RNA modifications modulate gene expression during development. *Science* 361 1346–1349. 10.1126/science.aau1646 30262497 PMC6436390

[B25] Galeano F. Tomaselli S. Locatelli F. Gallo A. (2012). A-to-I RNA editing: the “ADAR” side of human cancer. *Semin. Cell Dev. Biol.* 23 244–250. 10.1016/j.semcdb.2011.09.003 21930228

[B26] Guo Y. Zhao S. Sheng Q. Samuels D. C. Shyr Y. (2017). The discrepancy among single nucleotide variants detected by DNA and RNA high throughput sequencing data. *BMC Genomics* 18(Suppl. 6):690. 10.1186/s12864-017-4022-x 28984205 PMC5629567

[B27] Guryev V. Koudijs M. J. Berezikov E. Johnson S. L. Plasterk R. H. Van Eeden F. J. (2006). Genetic variation in the zebrafish. *Genome Res.* 16 491–497. 10.1101/gr.4791006 16533913 PMC1457036

[B28] Haussmann I. U. Bodi Z. Sanchez-Moran E. Mongan N. P. Archer N. Fray R. G. (2016). m6A potentiates Sxl alternative pre-mRNA splicing for robust Drosophila sex determination. *Nature* 540:301. 10.1038/nature20577 27919081

[B29] Heale B. S. Keegan L. P. O’connell M. A. (2010). “The effect of RNA editing and ADARs on miRNA biogenesis and function,” in *Regulation of microRNAs*, ed. Großhans H., (New York: Springer), 76–84. 10.1007/978-1-4419-7823-3_8

[B30] Heinz S. Benner C. Spann N. Bertolino E. Lin Y. C. Laslo P. (2010). Simple combinations of lineage-determining transcription factors prime cis-regulatory elements required for macrophage and B cell identities. *Mol. Cell* 38 576–589. 10.1016/j.molcel.2010.05.004 20513432 PMC2898526

[B31] Helm M. Motorin Y. (2017). Detecting RNA modifications in the epitranscriptome: predict and validate. *Nat. Rev. Genet.* 18 275–291. 10.1038/nrg.2016.169 28216634

[B32] Higuchi M. Maas S. Single F. N. Hartner J. Rozov A. Burnashev N. (2000). Point mutation in an AMPA receptor gene rescues lethality in mice deficient in the RNA-editing enzyme ADAR2. *Nature* 406 78–81. 10.1038/35017558 10894545

[B33] Hsu P. J. Zhu Y. Ma H. Guo Y. Shi X. Liu Y. (2017). Ythdc2 is an N6-methyladenosine binding protein that regulates mammalian spermatogenesis. *Cell Res.* 27 1115–1127. 10.1038/cr.2017.99 28809393 PMC5587856

[B34] Huang J. Yin P. (2018). Structural insights into N6-methyladenosine (m6A) modification in the transcriptome. *Genomics Proteomics Bioinformatics* 16 85–98. 29709557 10.1016/j.gpb.2018.03.001 PMC6112310

[B35] Hwang T. Park C.-K. Leung A. K. Gao Y. Hyde T. M. Kleinman J. E. (2016). Dynamic regulation of RNA editing in human brain development and disease. *Nat. Neurosci.* 19:1093. 10.1038/nn.4337 27348216

[B36] Ianniello Z. Paiardini A. Fatica A. (2019). N6-Methyladenosine (m6A): A promising new molecular target in acute myeloid leukemia. *Front. Oncol.* 9:251. 10.3389/fonc.2019.00251 31024852 PMC6465620

[B37] Ivanova I. Much C. Di Giacomo M. Azzi C. Morgan M. Moreira P. N. (2017). The RNA m6A reader YTHDF2 is essential for the post-transcriptional regulation of the maternal transcriptome and oocyte competence. *Mol. Cell* 67 1059.e4–1067.e4. 28867294 10.1016/j.molcel.2017.08.003 PMC5613143

[B38] Jean-Michel F. Masao D. Yoshiaki Y. Hayashi H. Shinichi N. Minoru Y. (2013). RNA-Methylation-Dependent RNA processing controls the speed of the circadian clock. *Cell* 155 793–806. 10.1016/j.cell.2013.10.026 24209618

[B39] Johnen H. González-Silva L. Carramolino L. Flores J. M. Torres M. Salvador J. M. (2013). Gadd45g is essential for primary sex determination, male fertility and testis development. *PLoS One* 8:e58751. 10.1371/journal.pone.0058751 23516551 PMC3596291

[B40] Kasowitz S. D. Ma J. Anderson S. J. (2018). Nuclear m6A reader YTHDC1 regulates alternative polyadenylation and splicing during mouse oocyte development. *PLoS Genet.* 14:e1007412. 10.1371/journal.pgen.1007412 29799838 PMC5991768

[B41] Kim D. Langmead B. Salzberg S. L. (2015). HISAT: a fast spliced aligner with low memory requirements. *Nat. Methods* 12:357. 10.1038/nmeth.3317 25751142 PMC4655817

[B42] Koslowsky D. J. (2004). *A historical perspective on RNA editing, in RNA Interference, Editing, and Modification.* Cham: Springer, 161–197. 10.1385/1-59259-775-0:161 15103074

[B43] Kowalewski M. P. Dyson M. T. Manna P. R. Stocco D. M. (2009). Involvement of peroxisome proliferator-activated receptor γ in gonadal steroidogenesis and steroidogenic acute regulatory protein expression. *Reprod. Fertil. Dev.* 21 909–922. 19698295 10.1071/RD09027

[B44] Lee H. N. Chang E. M. (2019). Primordial follicle activation as new treatment for primary ovarian insufficiency. *Clin. Exp. Reprod. Med.* 46 43–49. 10.5653/cerm.2019.46.2.43 31181871 PMC6572666

[B45] Lence T. Akhtar J. Bayer M. Schmid K. Spindler L. Ho C. H. (2016). m6A modulates neuronal functions and sex determination in *Drosophila*. *Nature* 540:242. 10.1038/nature20568 27919077

[B46] Li H. Liang Z. Yang J. Wang D. Wang H. Zhu M. (2019). DAZL is a master translational regulator of murine spermatogenesis. *Natl. Sci. Rev.* 6 455–468. 10.1093/nsr/nwy163 31355046 PMC6660020

[B47] Li X. Xiong X. Yi C. (2017). Epitranscriptome sequencing technologies: decoding RNA modifications. *Nat. Methods* 14 23–31. 10.1038/nmeth.4110 28032622

[B48] Liao S. Sun H. Xu C. (2018). YTH domain: a family of N~6-methyladenosine (m~6A) readers. *Genomics Proteomics Bioinformatics* 16 25–33. 10.1016/j.gpb.2018.04.002 PMC6112328 29715522

[B49] Lin Z. Hsu P. J. Xing X. Fang J. Lu Z. Zou Q. (2017). Mettl3-/Mettl14-mediated mRNA N6-methyladenosine modulates murine spermatogenesis. *Cell Res.* 27 1216–1230. 10.1038/cr.2017.117 28914256 PMC5630681

[B50] Linder B. Grozhik A. V. Olarerin-George A. O. Meydan C. Mason C. E. Jaffrey S. R. (2015). Single-nucleotide-resolution mapping of m6A and m6Am throughout the transcriptome. *Nat. Methods* 12 767–772. 10.1038/nmeth.3453 26121403 PMC4487409

[B51] Liu K. Rajareddy S. Liu L. Jagarlamudi K. Boman K. Selstam G. (2006). Control of mammalian oocyte growth and early follicular development by the oocyte PI3 kinase pathway: new roles for an old timer. *Dev. Biol.* 299 1–11. 10.1016/j.ydbio.2006.07.038 16970938

[B52] Liu N. Parisien M. Dai Q. Zheng G. He C. Pan T. (2013). Probing N6-methyladenosine RNA modification status at single nucleotide resolution in mRNA and long noncoding RNA. *RNA* 19 1848–1856. 10.1261/rna.041178.113 24141618 PMC3884656

[B53] Liu Q. Zhang Y. Shi B. Lu H. Zhang L. Zhang W. (2016). Foxo3b but not Foxo3a activates cyp19a1a in Epinephelus coioides. *J. Mol. Endocrinol.* 56 337–349. 10.1530/jme-15-0251 26960338

[B54] Martin M. (2011). Cutadapt removes adapter sequences from high-throughput sequencing reads. *EMBnet J.* 17 10–12.

[B55] Mauer J. Jaffrey S. R. (2018). FTO, m6Am, and the hypothesis of reversible epitranscriptomic mRNA modifications. *FEBS Lett.* 592 2012–2022. 10.1002/1873-3468.13092 29754392

[B56] McIntyre A. B. R. Gokhale N. S. Cerchietti L. Jaffrey S. R. Horner S. M. Mason C. E. (2020). Limits in the detection of m6A changes using MeRIP/m6A-seq. *Sci. Rep.* 10:6590. 10.1038/s41598-020-63355-3 PMC7170965 32313079

[B57] Mei J. Gui J. F. (2015). Genetic basis and biotechnological manipulation of sexual dimorphism and sex determination in fish. *Sci. China Life Sci.* 58 124–136. 10.1007/s11427-014-4797-9 25563981

[B58] Meng J. Lu Z. Liu H. Zhang L. Zhang S. Chen Y. (2014). A protocol for RNA methylation differential analysis with MeRIP-Seq data and exomePeak R/Bioconductor package. *Methods* 69 274–281. 10.1016/j.ymeth.2014.06.008 24979058 PMC4194139

[B59] Neaves W. B. Baumann P. (2011). Unisexual reproduction among vertebrates. *Trends Genet.* 27 81–88. 10.1016/j.tig.2010.12.002 21334090

[B60] Nishikura K. (2010). Functions and regulation of RNA editing by ADAR deaminases. *Annu. Rev. Biochem.* 79 321–349. 10.1146/annurev-biochem-060208-105251 20192758 PMC2953425

[B61] Nishikura K. (2016). A-to-I editing of coding and non-coding RNAs by ADARs. *Nat. Rev. Mol. Cell Biol.* 17 83–96. 10.1038/nrm.2015.4 26648264 PMC4824625

[B62] Oakes E. Anderson A. Cohen-Gadol A. Hundley H. A. (2017). Adenosine deaminase that acts on RNA 3 (ADAR3) binding to glutamate receptor subunit B pre-mRNA inhibits RNA editing in glioblastoma. *J. Biol. Chem.* 292 4326–4335. 10.1074/jbc.m117.779868 28167531 PMC5354488

[B63] Ortega-Recalde O. Goikoetxea A. Hore T. A. Todd E. V. Gemmell N. J. (2020). The genetics and epigenetics of sex change in fish. *Annu. Rev. Anim. Biosci.* 8 47–69. 10.1146/annurev-animal-021419-083634 31525067

[B64] Patil D. P. Pickering B. F. Jaffrey S. R. (2017). Reading m6A in the transcriptome: m6A-binding proteins. *Trends Cell Biol.* 28 113–127. 10.1016/j.tcb.2017.10.001 29103884 PMC5794650

[B65] Peng Z. Cheng Y. Tan B. C.-M. Kang L. Tian Z. Zhu Y. (2012). Comprehensive analysis of RNA-Seq data reveals extensive RNA editing in a human transcriptome. *Nat. Biotechnol.* 30 253–260. 10.1038/nbt.2122 22327324

[B66] Pertea M. Pertea G. M. Antonescu C. M. Chang T.-C. Mendell J. T. Salzberg S. L. (2015). StringTie enables improved reconstruction of a transcriptome from RNA-seq reads. *Nat. Biotechnol.* 33 290–295. 10.1038/nbt.3122 25690850 PMC4643835

[B67] Pfennig F. Standke A. Gutzeit H. O. (2015). The role of Amh signaling in teleost fish-multiple functions not restricted to the gonads. *Gen. Comp. Endocrinol.* 223 87–107. 10.1016/j.ygcen.2015.09.025 26428616

[B68] Picardi E. Manzari C. Mastropasqua F. Aiello I. D’erchia A. M. Pesole G. (2015). Profiling RNA editing in human tissues: towards the inosinome Atlas. *Sci. Rep.* 5:14941. 10.1038/srep14941 PMC4598827 26449202

[B69] Piferrer F. (2018). “Epigenetics of sex determination and differentiation in fish,” in *Sex Control in Aquaculture*, eds Wang H. P. Piferrer F. Chen S. L. Shen Z. G., (Hoboken, NJ: Wiley), 65–83. 10.1002/9781119127291.ch3

[B70] Piprek R. P. Podkowa D. Kloc M. Kubiak J. Z. (2019). Expression of primary cilia-related genes in developing mouse gonads. *Int. J. Dev. Biol.* 63 615–621. 10.1387/ijdb.190049rp 32149371

[B71] Qi S. T. Ma J. Y. Wang Z. B. Guo L. Hou Y. Sun Q. Y. (2016). N6-methyladenosine sequencing highlights the involvement of mRNA methylation in oocyte meiotic maturation and embryo development by regulating translation in *Xenopus laevis*. *J. Biol. Chem.* 291 23020–23026. 10.1074/jbc.m116.748889 27613873 PMC5087722

[B72] Richburg J. H. Myers J. L. Bratton S. B. (2014). The role of E3 ligases in the ubiquitin-dependent regulation of spermatogenesis. *Semin. Cell Dev. Biol.* 30 27–35. 10.1016/j.semcdb.2014.03.001 24632385 PMC4043869

[B73] Robinson M. D. Mccarthy D. J. Smyth G. K. (2010). edgeR: a Bioconductor package for differential expression analysis of digital gene expression data. *Bioinformatics* 26 139–140. 10.1093/bioinformatics/btp616 19910308 PMC2796818

[B74] Roundtree I. A. Evans M. E. Pan T. He C. (2017). Dynamic RNA modifications in gene expression regulation. *Cell* 169 1187–1200. 10.1016/j.cell.2017.05.045 28622506 PMC5657247

[B75] Si Y. Ding Y. He F. Wen H. Li J. Zhao J. (2016). DNA methylation level of cyp19a1a and Foxl2 gene related to their expression patterns and reproduction traits during ovary development stages of Japanese flounder (*Paralichthys olivaceus*). *Gene* 575 321–330. 10.1016/j.gene.2015.09.006 26343797

[B76] Slotkin W. Nishikura K. (2013). Adenosine-to-inosine RNA editing and human disease. *Genome Med.* 5:105. 10.1186/gm508 24289319 PMC3979043

[B77] Snyder E. M. Licht K. Braun R. E. (2017). Testicular adenosine to inosine RNA editing in the mouse is mediated by ADARB1. *Biol. Reprod.* 96 244–253. 28395340 10.1095/biolreprod.116.145151 PMC6279061

[B78] Sun P. You F. Liu M. Wu Z. Wen A. Li J. (2010). Steroid sex hormone dynamics during estradiol-17β induced gonadal differentiation in *Paralichthys olivaceus* (Teleostei). *Chinese J. Oceanol. Limnol.* 28 254–259. 10.1007/s00343-010-9011-8

[B79] Sun P. You F. Zhang L. J. Wen A. Y. Wu Z. H. Xu D. D. (2009). Histological evaluation of gonadal differentiation in olive flounder (*Paralichthys olivaceus*). *Mar. Sci.* 33 53–58.

[B80] Taisuke H. Biggs W. H. David T. Boyer A. D. Varki N. M. Cavenee W. K. (2004). Disruption of forkhead transcription factor (FOXO) family members in mice reveals their functional diversification. *Proc. Natl. Acad. Sci. U.S.A.* 101 2975–2980. 10.1073/pnas.0400093101 14978268 PMC365730

[B81] Tajaddod M. Jantsch M. F. Licht K. (2016). The dynamic epitranscriptome: A to I editing modulates genetic information. *Chromosoma* 125 51–63. 10.1007/s00412-015-0526-9 26148686 PMC4761006

[B82] Tan M. H. Li Q. Shanmugam R. Piskol R. Kohler J. Young A. N. (2017). Dynamic landscape and regulation of RNA editing in mammals. *Nature* 550 249–254. 29022589 10.1038/nature24041 PMC5723435

[B83] Tao H. Qiong W. Guihai F. Yaou H. Li W. Yumin W. (2012). Computational detection and functional analysis of human tissue-specific A-to-I RNA editing. *PLoS One* 6:e18129. 10.1371/journal.pone.0018129 21448465 PMC3063316

[B84] Wainwright E. N. Svingen T. Ng E. T. Wicking C. Koopman P. (2014). Primary cilia function regulates the length of the embryonic trunk axis and urogenital field in mice. *Dev. Biol.* 395 342–354. 10.1016/j.ydbio.2014.08.037 25224227

[B85] Wang Q. Khillan J. Gadue P. Nishikura K. (2000). Requirement of the RNA editing deaminase ADAR1 gene for embryonic erythropoiesis. *Science* 290 1765–1768. 10.1126/science.290.5497.1765 11099415

[B86] Wang W. Liang S. Zou Y. Wu Z. Wang L. Liu Y. (2020). Amh dominant expression in Sertoli cells during the testicular differentiation and development stages in the olive flounder *Paralichthys olivaceus*. *Gene* 755:144906. 10.1016/j.gene.2020.144906 32554048

[B87] Wang X. Wu X. Zhu Z. Li H. Li T. Li Q. (2019). Landscape of RNA editing reveals new insights into the dynamic gene regulation of spermatogenesis. *Cell Cycle* 18 3351–3364. 10.1080/15384101.2019.1676584 31594448 PMC6927729

[B88] Wang Y. Xu X. Yu S. Kang K. J. Zhou Z. Han L. (2017). Systematic characterization of A-to-I RNA editing hotspots in microRNAs across human cancers. *Genome Res.* 27 1112–1125. 10.1101/gr.219741.116 28411194 PMC5495064

[B89] Wang Z. Lian J. Li Q. Zhang P. Zhou Y. Zhan X. (2016). RES-Scanner: a software package for genome-wide identification of RNA-editing sites. *GigaScience* 5;37. 10.1186/s13742-016-0143-4 PMC4989487 27538485

[B90] Webster K. A. Schach U. Ordaz A. Steinfeld J. S. Draper B. W. Siegfried K. R. (2017). Dmrt1 is necessary for male sexual development in zebrafish. *Dev. Biol.* 422 33–46. 10.1016/j.ydbio.2016.12.008 27940159 PMC5777149

[B91] Wen A. You F. Sun P. Li J. Xu D. Wu Z. (2014). CpG methylation of dmrt1 and cyp19a promoters in relation to their sexual dimorphic expression in the Japanese flounder *Paralichthys olivaceus*. *J. Fish Biol.* 84 193–205. 10.1111/jfb.12277 24372528

[B92] Xia H. Zhong C. Wu X. Chen J. Tao B. Xia X. (2018). Mettl3 mutation disrupts gamete maturation and reduces fertility in zebrafish. *Genetics* 208 729–743. 10.1534/genetics.117.300574 29196300 PMC5788534

[B93] Xiang J. F. Yang Q. Liu C. X. Wu M. Chen L. L. Yang L. (2018). N6-Methyladenosines modulate A-to-I RNA editing. *Mol. Cell* 69 126–135. 29304330 10.1016/j.molcel.2017.12.006

[B94] Xu K. Yang Y. Feng G. H. Sun B. F. Chen J. Q. Li Y. F. (2017). Mettl3-mediated m6A regulates spermatogonial differentiation and meiosis initiation. *Cell Res.* 27 1100–1114. 10.1038/cr.2017.100 28809392 PMC5587845

[B95] Yamamoto E. (1999). Studies on sex-manipulation and production of cloned populations in hirame, *Paralichthys olivaceus* (Temminck et Schlegel). *Aquaculture* 173 235–246. 10.1016/s0044-8486(98)00448-7

[B96] Yang D. Yin C. Chang Y. Dou Y. Hao Z. Ding J. (2016). Transcriptome analysis of male and female mature gonads of Japanese scallop Patinopecten yessonsis. *Genes Genomics* 38 1041–1052. 10.1007/s13258-016-0449-8

[B97] Yang H. Xiaobin L. Ting L. Jia S. Wang H. Song G. (2015). Expression and significance of SR-protein-specific kinase SRPK2 in mouse testis. *Acta Lab. Anim. Sci. Sin.* 23 171–177.

[B98] Yazawa T. Inaoka Y. Okada R. Mizutani T. Yamazaki Y. Usami Y. (2010). PPAR-γ coactivator-1α regulates progesterone production in ovarian granulosa cells with SF-1 and LRH-1. *Mol. Endocrinol.* 24 485–496. 10.1210/me.2009-0352 20133449 PMC5419099

[B99] Yen P. H. (2004). Putative biological functions of the DAZ family. *Int. J. Androl.* 27 125–129. 10.1111/j.1365-2605.2004.00469.x 15139965

[B100] You F. Liu J. Wang X. C. Xu Y. L. Huang R. D. Zhang P. J. (2001). Study on embryonic development and early growth of triploid and gynogenetic diploid left-eyed flounder, *Paralichthys olivaceus* (T. et S.). *Chinese J. Oceanol. Limnol.* 19 147–151. 10.1007/bf02863039

[B101] Yu G. Wang L. G. He Q. Y. (2015). ChIPseeker: an R/Bioconductor package for ChIP peak annotation, comparison and visualization. *Bioinformatics* 31 2382–2383. 10.1093/bioinformatics/btv145 25765347

[B102] Zeng S. Gong Z. (2002). Expressed sequence tag analysis of expression profiles of zebrafish testis and ovary. *Gene* 294 45–53. 10.1016/s0378-1119(02)00791-6 12234666

[B103] Zhang C. Chen Y. Sun B. Wang L. Yang Y. Ma D. (2017). m6A modulates haematopoietic stem and progenitor cell specification. *Nature* 549:273. 10.1038/nature23883 28869969

[B104] Zhang Y. Zhang L. Yue J. Wei X. Wang L. Liu X. (2019). Genome-wide identification of RNA editing in seven porcine tissues by matched DNA and RNA high-throughput sequencing. *J. Anim. Sci. Biotechnol.* 10:24. 10.1186/s40104-019-0326-9 PMC6415349 30911384

[B105] Zheng G. Dahl J. A. Niu Y. Fedorcsak P. Huang C.-M. Li C. J. (2013). ALKBH5 is a mammalian RNA demethylase that impacts RNA metabolism and mouse fertility. *Mol. Cell* 49 18–29. 10.1016/j.molcel.2012.10.015 23177736 PMC3646334

[B106] Zheng W.-J. Sun L. (2011). Evaluation of housekeeping genes as references for quantitative real time RT-PCR analysis of gene expression in Japanese flounder (*Paralichthys olivaceus*). *Fish Shellfish Immunol.* 30 638–645. 10.1016/j.fsi.2010.12.014 21185941

[B107] Zhong Q. Zhang Q. Chen Y. Sun Y. Qi J. Wang Z. (2008). The isolation and characterization of myostatin gene in Japanese flounder (*Paralichthys olivaceus*): ubiquitous tissue expression and developmental specific regulation. *Aquaculture* 280 247–255. 10.1016/j.aquaculture.2008.04.015

